# Associations between adverse childhood experiences and pain in middle-aged and older adults: findings from the China Health and Retirement Longitudinal Study

**DOI:** 10.1186/s12889-024-19239-6

**Published:** 2024-07-02

**Authors:** Jing Luo, Yue Ma, Hao-Wei Zhan, Wang-Hu Jia, Jia-Rui Zhang, Shi-Yu Xie, Si-Yin Yu, Shuang-Long Hou, Xia Bi, Xue-Qiang Wang

**Affiliations:** 1 Rehabilitation Medicine Center, The Second Affiliated Hospital of Wenzhou Medical University, Wenzhou, 325027 Zhejiang China; 2 Department of Sport Rehabilitation, Xi’an Physical Education University, Xi’an, 710068 China; 3 Department of Rehabilitation Medicine, Shanghai University of Medicine and Health Sciences Affiliated Zhoupu Hospital, Shanghai, China; 4 Department of Sport Rehabilitation, Shanghai University of Sport, Shanghai, 200438 China; 5 School of Rehabilitation Medicine, Wenzhou Medical University, Wenzhou, 325035 Zhejiang China

**Keywords:** Pain, Adverse childhood experiences, Physical activity, China

## Abstract

**Objective:**

Adverse childhood experiences (ACEs) have been associated with a range of adverse health outcomes, with pain being potentially one of them. This population-based cross-sectional study aimed to investigate the associations between Adverse Childhood Experiences (ACEs) and pain in Chinese adults and evaluate whether physical activity and demographic and socioeconomic characteristics modify this associations.

**Methods:**

Cross-sectional data from the China Health and Retirement Longitudinal Study (CHARLS), were utilized in this study. A total of 9923 respondents with information on 12 ACE indicators and 15 self-reported body pains were included. Logistic regression models were used to assess associations of the ACEs and pain. Modification of the associations by physical activity, demographic and socioeconomic characteristics was assessed by stratified analyses and tests for interaction.

**Results:**

Among the 9923 individuals included in the primary analyses, 5098 (51.4%) males and the mean (SD) age was 61.18 (10·.44) years. Compared with individuals with 0 ACEs, those who with ≥ 5 ACEs had increased risk of single pains and multiple pain. A dose–response association was found between the number of ACEs and the risk of pain (e.g. neck pain for ≥ 5 ACEs vs. none: OR, 1.107; 95% CI, 0.903–1.356; *p* < 0.001 for trend). In the associations of each body pain with each ACE indicator, most ACE indicators were associated with an increased risk of pain. In addition, physical activity, sociodemographic and socioeconomic characteristics, such as age, sex, educational level, area of residence, childhood economic hardship, did not demonstrate a significant modify on the associations between ACEs and pain.

**Conclusions:**

These findings indicate that cumulative ACE exposure is associated with increased odds of self-reported pain in Chinese adults, regardless of adult physical activity, sociodemographic and socioeconomic characteristics.

**Supplementary Information:**

The online version contains supplementary material available at 10.1186/s12889-024-19239-6.

## Introduction

Adverse childhood experiences (ACEs) refer to intense and potentially stressful experiences during childhood. In the past 2 decades, many researchers examined the prevalence of ACEs and a range of poor health and quality-of-life outcomes [1, 2]. More than 1 billion children were estimated to be exposed to violence globally in 2018 alone [3]. In Europe, the prevalence of one ACE is 23.5%, and that of two or more ACEs is 18.7%; in North America, these figures are 23.4% and 35%, respectively [2, 4]. ACEs also represent a significant healthcare burden, with the total annual cost of ACEs being over $1329 billion in Europe and North America, and the lifetime economic burden of ACE abuse alone being $585 billion [2, 5]. Although ACEs may impose significant healthcare burdens, much of the research has been conducted in developed countries. Theoretically, ACEs may be more prevalent in developing countries like China [6, 7], yet there are few studies reporting on this.

The poor health outcomes caused by ACEs have large extension, breadth, and long-term effects. Many researches explored the associations between ACEs and deleterious consequences for health such as cardiovascular disease [8], dementia [9], cognitive decline, and digestive disease [10]. Some physiological and biomolecular studies indicated how childhood exposure to chronic stress leads to changes in cardio/metabolic systems, genetics, and inflammation, resulting in related disease [11–13]. Pain is one of the most popular reasons for adults to seek medical care.[14] However, the associations of ACEs with pain has not been fully explored.

Many studies investigated potential effect modifiers that may modify the associations between ACEs and adverse health outcomes [10, 15]. These factors can be explored to identify effective interventions to decrease risks of pain originating from exposure to ACEs. In recent years, exercise has become one of the main treatments for pain [16]. Abundant research findings suggest that physical activity yields notable analgesic effects on pain [17, 18], and demographic and socioeconomic characteristics are frequently addressed as to whether they are associated with adverse health outcomes related to ACEs [19]. Therefore, exploring the modifying role of physical activity and demographic and socioeconomic factors in the associations between ACEs and pain is necessary.

In this study, data from the China Health and Retirement Longitudinal Study (CHARLS) were used to investigate the associations between ACEs and pain in Chinese adults. The modifying role of physical activity and demographic and socioeconomic characteristics was evaluated.

## Methods

### Study design and population

This study used data from CHARLS, an ongoing and nationally representative longitudinal survey initiated in 2011. CHARLS encompassed individuals across 150 counties or districts and 450 villages or urban communities in 28 Chinese provinces. This extensive survey employed a multistage stratified sampling approach that was based on probability-proportionate-to-size principles. To date, the CHARLS project carried out three subsequent surveys in 2013, 2015, and 2018. The comprehensive research framework and selection techniques were previously documented and disclosed [20]. In the present study, data from the 2014 life history survey and the subsequent 2015 follow-up survey, which were carried out between June 1, 2014, and December 31, 2014, and from July 1, 2015, to September 30, 2015, were utilized. Each participant provided explicit consent, and CHARLS obtained ethical approval, from the Institutional Review Board of Peking University (number: IRB00001052-11015) [20]. The study followed the reporting principles specified in the Strengthening the Reporting of Observational Studies in Epidemiology (STROBE) checklist (Supplementary Material).

**Fig. 1:**
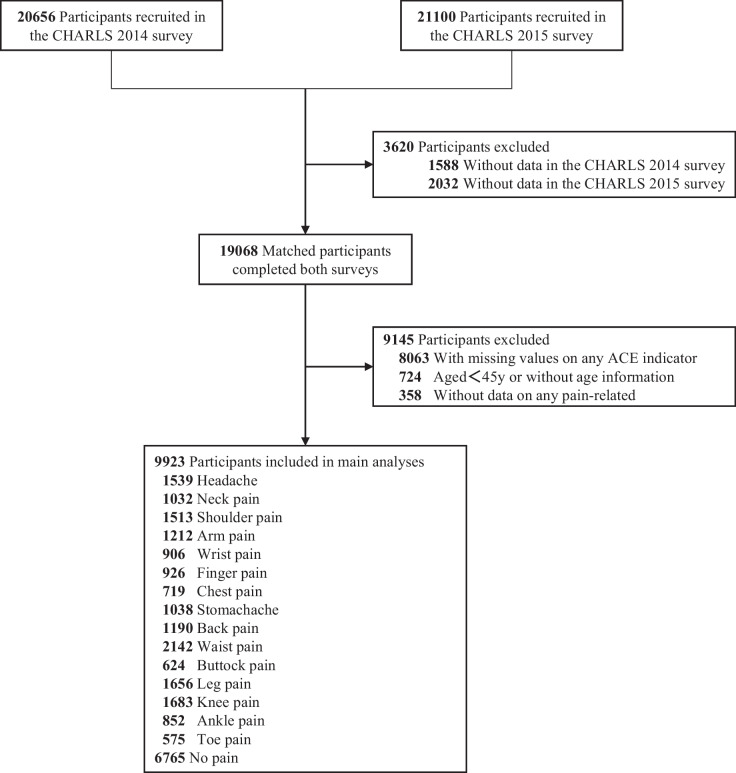
Flow-chart of the Analytic Sample

A successful 1:1 matching process for 19,068 participants selected from the pool of 20,656 individuals from the 2014 life history survey and 21,100 subjects from the 2015 follow-up survey who completed both surveys was accomplished. And the life history survey conducted in 2014 collected data related to ACE from the participants, while the data survey in 2015 collected other data besides ACE data. The inclusion criteria were as follows: 1) participants in 2014 CHARLS survey and 2) participants in 2015 CHARLS survey. The exclusion criteria were as follows: 1) with missing data of ACEs (*n* = 8063); 2) aged < 45 years or lack of age information (*n* = 724); and 3) lack of any pain-related data (*n* = 358). The total of 9923 individuals who matched the criteria were finally included for the main statistical analysis. A comprehensive depiction of the selection procedure is presented in Fig. 1.

### Definitions of pain

The primary outcomes in this study were 15 self-reported body pains for which the data were collected from 2014 and 2015 CHARLS follow-up surveys. Headache, neck pain, shoulder pain, arm pain, wrist pain, finger pain, chest pain, stomachache, back pain, waist pain, buttock pain, leg pain, knee pain, ankle pain, and toe pain were defined on the basis of individuals’ self-report and how often they were troubled by these body pains. The secondary outcome was multiple pain, which was characterized by simultaneous occurrence of 2 or more out of the 15 body pains in the same individual [21]. Participants without any body pain or with only one body pain were categorized into non-multiple pain group.

### Definition of adverse childhood experiences

In accordance with the detailed questionnaire items from the CHARLS dataset, 12 ACEs were extracted, including original ACEs [22, 23] expanded ACEs [4], and new reported ACEs in recent years [19, 24]. The specific questionnaire items and definition of ACEs are available in Table S1. Each ACE indicator was dichotomized, encoded (0 for absent or 1 for present), and summed to derive the cumulative scores of ACEs for each individual on the basis of their responses to the relevant ACE questionnaire items. In accordance with the cumulative ACE scores, the participants were categorized into 6 groups: 0, 1, 2, 3, 4, and ≥ 5.

### Covariates

Covariates, including sex, age, marital status, smoking or not, drinking status, obesity, childhood family economic hardship, area of residence, chronic diseases, education, and physical activity were obtained through in-person interview.

In addition to the covariates commonly used in demographic characteristics [25], we also included variables related to socioeconomic characteristics, such as childhood family economic hardship, which is considered a good indicator of childhood economic status [26]. The risk of some chronic diseases has recently been reported to have a dose–response association with the number of ACEs [25], but whether some chronic diseases affect the associations between pain and ACEs has not been explored. Furthermore, we are highly interested in whether physical activity modifies this associations, as exercise therapy for pain has garnered significant attention from researchers and clinicians in recent decades [27], and is recommended by many international guidelines [28]. And in this study, physical activity data were generated through self-reporting of exercise duration for three levels of physical activity intensity: vigorous physical activities (VPA), moderate physical activities (MPA), low physical activities (LPA).

Among them, chronic disease status was determined by the participant’s self-reported diagnosis by a doctor or in conjunction with health evaluations and medication records collected during the 2015 CHARLS survey. Details on the three types of physical activities performed in the past week were collected. Additional information about these covariates can be found in Supplement.

### Statistical analysis

Chi-square test was used for categorical data, and ANOVA was used for continuous data for comparisons and description of the characteristics. Polynomial comparisons were employed to analyze variance in trends for continuous data, while the Mantel–Haenszel statistic was utilized for categorical data to assess trends in characteristics across different ACE groups.

Four models were constructed using logistic regression to explore the associations between cumulative ACE score and 15 body pains and the hypotheses of the logistic regression models were as follows: (1) experiencing 5 or more ACEs was associated with 15 self-reported body pains compared with no ACE exposure. (2) significant dose–response association between cumulative ACEs cores and body pains. Model 1 is a crude model of 15 body pains and 6 ACE groups. Model 2 is adjusted for age, sex, and body mass index (BMI). Model 3 is adjusted for residence, educational level, marital status, residence, smoking, drinking, childhood economic hardship, and physical activities on the basis of model 2. Model 4 is the fully adjusted model that adjusted 14 chronic diseases on the basis of model 3. All regression models reported the odds ratios (ORs), 95% CIs, and *p* trend of different ACE groups. Regression analyses of each ACE (prevalence ≥ 0.1) with pain were conducted to further assess the associations of pain with ACEs. The associations between pain and ACEs within the overall population and subgroups (sex, age, childhood family economic hardship, area of residence, educational level, and physical activities) was further examined to determine the potential modifying factors. Fifty imputed datasets utilizing chained Eqs [29], were created, and then select the most reliable dataset. The outcomes of all regression models were re-analyzed to comprehensively account for missing data.

Data were analyzed from May 2023, to September 2023. Primary statistical analyses were conducted using Stata (version 15.0, StataCorp, LLC). Two-sided *P* < 0.05 was considered statistically significant.

## Results

### Characteristics of participants

The primary analysis involved 9923 participants, with 5098 (51.4%) males and 4825 (48.6%) females. The mean (SD) age was 61.18 (10.44) years. Overall, 2288 (23.1%) participants had 0 exposure to ACEs, 3104 (31.3%) had one exposure to ACE, 2266 (22.8%) had two exposures to ACEs, 1315 (13.3%) had three exposures to ACEs, 605 (6.1%) had four exposures to ACEs, and 345 (3.5%) had five or more exposures to ACEs. Table 1 presents the descriptive statistics for the study population. The participants with higher ACEs scores (ACEs ≥ to 3) were more likely to be married, live in a rural area, have had childhood family economic hardship, and smoke than those with no ACEs. Of the 12 ACEs, the lowest prevalence rate was found in incarcerated household members (0.28%), whereas the highest was observed in physical abuse (18.61%, Table S1).

**Table 1:** Characteristics of Participants by Number of ACEs

Characteristics^a^	ACE, No						*P*-value	*P*-value for trend
	**0(*n* = 2288)**	**1(*n* = 3104)**	**2(*n* = 2266)**	**3(*n* = 1315)**	**4(*n* = 6050**	** ≥ 5(*n* = 345)**		
**Ages, y**	61.11(10.65)	61.40(10.42)	61.16(10.50)	60.80(10.07)	61.30(10.38)	60.69(10.14)	0.102	0.217
**Sex**								
Male	1108(48.4%)	1610(51.9%)	1193(52.6%)	703(53.5%)	307(50.7%)	177(51.3%)	0.330	0.035
Female	1180(51.6%)	1494(48.1%)	1073(47.4%)	612(46.5%)	298(49.3%)	168(48.7%)		
**Marital status**								
Married	1679(73.4%)	2251(72.5%)	1604(70.8%)	958(72.9%)	413(68.3%)	234(67.8%)	0.037	0.007
Unmarried	609(26.6%)	853(27.5%)	662(29.2%)	357(27.1%)	192(31.7%)	111(32.2%)		
**Educational level completed**								
None	429(18.8%)	575(18.5%)	436(19.2%)	261(19.8%)	131(21.7%)	69(20.0%)	0.001	< 0.001
Home school to primaryschool	830(36.3%)	1261(40.6%)	945(41.7%)	600(45.6%)	271(44.8%)	169(49.0%)		
Middle above	1026(44.9%)	1268(40.9%)	885(39.1%)	454(34.5%)	203(33.6%)	107(31.0%)		
**Area of residence**								
Rural	1696(74.3%)	2356(76.2%)	1717(75.8%)	1015(77.8%)	473(78.4%)	263(76.9%)	0.150	0.015
Urban	586(25.7%)	736(23.8%)	544(24.1%)	289(22.2%)	130(21.6%)	79(23.1%)		
**Childhood family economic hardship**								
No	1625(71.8%)	1091(35.3%)	1266(56.0%)	633(48.2%)	259(43.0%)	118(34.2%)	0.001	< 0.001
Yes	638(28.2%)	2002(64.7%)	993(44.0%)	679(51.8%)	344(57.0%)	227(65.8%)		
**Smoke status**								
Still smoke	583(63.8%)	757(62.4%)	546(65.0%)	325(64.2%)	148(63.2%)	84(63.6%)	0.893	0.981
Quit	269(29.4%)	371(30.6%)	240(28.6%)	153(30.2%)	68(29.1%)	39(29.5%)		
Never smoke	62(6.8)	85(7.0%)	54(6.4%)	28(5.5%)	18(7.7)	9(6.8%)		
**Drink status**								
Never had a drink	1337(76.7%)	1701(75%)	1163(72.4%)	652(68.3%)	320(74.4%)	152(61.8%)	0.001	< 0.001
≤ 1/month	187(10.7%)	283(12.5%)	206(12.8%)	148(15.5%)	43(10.0%)	34(13.8%)		
> 1/month	219(12.6%)	285(12.6%)	238(14.8%)	154(16.1%)	67(15.6%)	60(24.4%)		
**Obesity**^b^								
Yes	277(18.3%)	326(12.6%)	233(12.3%)	141(12.6%)	64(12.3%)	40(13.0%)	0.290	0.378
No	1614(69.7%)	2258(87.4%)	1666(87.7%)	978(87.4%)	457(87.7%)	268(87.0%)		
**Chronic diseases**								
Hypertension	502(21.9%)	734(23.6%)	535(23.6%)	314(23.9%)	128(21.2%)	67(19.4%)	0.229	0.714
Dyslipidemia	198(8.7%)	318(10.2%)	225(9.9%)	138(10.5%)	58(9.6%)	35(10.1%)	0.424	0.215
Diabetes	126(5.5%)	153(4.9%)	130(5.7%)	88(6.7%)	33(5.5%)	28(8.1%)	0.080	0.033
Cancer	29(1.3%)	25(0.8%)	16(0.7%)	7(0.5%)	6(0.9%)	7(2.0%)	0.040	0.652
Chronic lung diseases	212(9.3%)	298(9.6%)	267(11.8%)	182(13.8%)	80(13.2%)	62(17.97%)	< 0.001	< 0.001
Liver disease	83(3.6%)	120(3.9%)	101(4.5%)	71(5.4%)	46(7.6%)	24(6.96%)	< 0.001	< 0.001
Heart attack	263(11.5%)	353(11.4%)	288(12.7%)	172(13.1%)	75(28.9%)	47 (13.6%)	0.402	0.058
Stroke	38(1.7%)	76(2.4%)	53(2.3%)	40(3.0%)	13(2.1%)	8(2.3%)	0.169	0.099
Kidney disease	114(4.98%)	191(6.2%)	161(7.1%)	116(8.8%)	62(10.2%)	39(11.3%)	< 0.001	< 0.001
Stomachache	418(18.3%)	670(21.6%)	569(25.1%)	348(26.5%)	175(28.9%)	120(34.8%)	< 0.001	< 0.001
Psychiatric disease	17(0.7%)	26(0.8%)	30(1.4%)	16(1.2%)	10(1.7%)	6(1.7%)	0.108	0.006
Memory-related disease	31(1.4%)	39(1.3%)	31(1.4%)	21(1.6%)	11(1.8%)	6(1.7%)	0.858	0.253
Arthritis	672(29.4%)	1016(32.7%)	868(38.3%)	538(40.9%)	260(43.0%)	169(49.0%)	< 0.001	< 0.001
Asthma	75(3.3%)	103(3.3%)	117(5.2%)	78(5.9%)	33(5.5%)	24(7.0%)	< 0.001	< 0.001
**Physical activities**								
No activity	151(6.6%)	145(4.7%)	112(4.9%)	67(5.1%)	26(4.3%)	17(4.9%)	0.383	0.232
With activity	971(42.4%)	1379(44.4%)	1034(45.6%)	585(44.5%)	268(44.3%)	163(47.2%)		
**With activities**								
** VPA**	333(29.6%)	494(32.4%)	406(35.4%)	241(37.1%)	124(42.2%)	70 (38.7%)		
10-74min/wk	6(0.5%)	12(0.8%)	4(0.3%)	7(1.1%)	1(0.3%)	2(1.1%)	< 0.001	< 0.001
75-244min/wk	40(3.6%)	68(4.5%)	65(5.7%)	30(4.6%)	9(3.1%)	7(3.9%)		
≥ 300min/wk	287(25.5%)	415(27.2%)	337(29.4%)	204(31.4%)	114(38.8%)	61(33.7%)		
** MPA**	607(54.1)	810(53.2%)	620(54.3%)	368(57.1%)	164(55.6%)	105(57.7%)		
10-149min/wk	93(8.3%)	134(8.8%)	77(6.7%)	62(9.6%)	16(5.4%)	10(5.5%)	0.078	0.140
150-299min/wk	63(5.6%)	77(5.1%)	45(3.9%)	27(4.2%)	12(4.1%)	12(6.6%)		
≥ 300min/wk	451(40.2%)	599(39.4%)	498(43.6%)	279(43.3%)	136(46.1%)	83(45.6%)		
** LPA**	842(75.3%)	1201(79.1%)	920(81.1%)	514(79.3%)	244(82.7%)	149(83.2%)		
10-140min/wk	133(11.9%)	192(12.6%)	148(13.1%)	78(12.0%)	43(14.6%)	19(10.6%)	0.025	0.001
141-525min/wk	398(35.6%)	574(37.8%)	426(37.6%)	231(35.6%)	104(35.3%)	67(37.4%)		
526-1260min/wk	214(19.1%)	263(17.3%)	203(17.9%)	133(20.5%)	53(18.0%)	37(20.7%)		
> 1260min/wk	97(8.7%)	172(11.3%)	143(12.6%)	72(11.1%)	44(14.9%)	26(14.5%)		

*Abbreviations*: *ACE* adverse childhood experience, *BMI* body mass index (calculated as weight in kilograms divided by height in meters squared), *VPA* Vigorous Physical Activities, *MPA* Moderate Physical Activities, *LPA* Low Physical Activities

^a^Continuous data are reported as the mean (SD), and categorical data are reported as the number and percentage of participants

^b^Defined as a BMI of 28 or greater

### Associations between ACEs and pain

Table 2 provides the prevalence of pain by number of ACEs, and we observed that an increase in the number of ACEs was associated with an increasing trend in the prevalence of pain. The logistic regression models revealed the associations of ACEs with these body pains. Overall, 5 or more ACEs (relative to 0 ACE) were associated with associated with a higher risk of 15 self-reported body pains. The adjusted ORs ranged from 2.555 (95% CI, 1.842–3.545) for back pain to 3.881 (95% CI, 2.662–5.659) for chest pain in adjusted model 2; 1.690 (95% CI, 0.750–3.806) for back pain to 3.740 (95% CI, 1.805–7.600) for finger pain in adjusted model 3;1.283 (95% CI, 0.495–3.329) for buttock pain to 3.069 (95% CI, 1.433–6.570) for finger pain in fully adjusted model and we also observed the same dose–response association between cumulative ACE scores with body pains(except stomachache and leg pain) as in the crude model(Table 3). There is no potential underlying collinearity between the independent variables included in the regression models. After multiple imputation was applied to address the missing data, an increase in the risk of some pain was found in the ACE groups, especially in fully adjusted model (Table S2).

**Table 2:** Prevalence of pain by Number of ACEs

Characteristics	ACE, No						*P*-value	*P*-value for trend
	**0(*n* = 2288)**	**1(*n* = 3104)**	**2(*n* = 2266)**	**3(*n* = 1315)**	**4(*n* = 6050**	**≥ 5(*n* = 345)**		
pain = 0	1737(75.9%)	2246(72.4%)	1526(67.3%)	825(62.7%)	335(55.4%)	159(46.1%)	< 0.001	< 0.001
pain = 1(Single pain)	94(4.1%)	136(4.4%)	104(4.6%)	56(4.3%)	37(6.1%)	23(6.7%)		
pain = 2	68(3.0%)	120(3.9%)	91(4.0%)	64(4.9%)	35(5.8%)	27(7.8%)		
Pain ≥ 2(Multiple pain)	457(20.0%)	722(23.3%)	636(28.1%)	434(33.0%)	233(38.5%)	163(47.3%)		
pain ≥ 3	389(17.0%)	602(19.4%)	545(24.1%)	370(28.1%)	198(32.8%)	136(39.4%)		
Headache	264(11.5%)	418(13.5%)	365(16.1%)	266(20.2%)	133(22.0%)	97(28.1%)		
Neck pain	180(7.9%)	278(9.0%)	248(10.9%)	163(12.4%)	93(15.4%)	70(20.3%)		
Shoulder pain	270(11.8%)	403(13.0%)	363(16.0%)	250(19.0%)	133(22.0%)	95(27.5%)		
Arm pain	199(8.7%)	325(10.5%)	310(13.7%)	199(15.1%)	98(16.2%)	82(23.8%)		
Wrist pain	145(6.3%)	248(8.0%)	231(10.2%)	146(11.1%)	75(12.4%)	61(17.7%)		
Finger pain	158(6.9%)	255(8.2%)	232(10.2%)	141(10.7%)	74(12.2%)	66(19.1%)		
Chest pain	111(4.9%)	200(6.4%)	165(7.3%)	119(9.0%)	69(11.4%)	56(16.2%)		
Stomachache	168(7.3%)	290(9.3%)	229(10.1%)	180(13.7%)	98(16.2%)	73(21.2%)		
Back pain	204(8.9%)	342(11.0%)	281(12.4%)	183(13.9%)	113(18.7%)	68(19.7%)		
Waist pain	368(16.1%)	579(18.7%)	521(23.0%)	351(26.7%)	192(31.7%)	131(38.0%)		
Buttock pain	109(4.8%)	157(5.1%)	165(7.3%)	103(7.8%)	46(7.6%)	44(12.8%)		
Leg pain	297(13.0%)	451(14.5%)	421(18.6%)	248(18.9%)	136(22.5%)	103(29.9%)		
Knee pain	301(13.2%)	441(14.2%)	422(18.6%)	275(20.9%)	141(23.3%)	103(29.9%)		
Ankle pain	137(6.0%)	216(7.0%)	220(9.7%)	132(10.0%)	84(13.9%)	63(18.3%)		
Toe pain	97(4.2%)	162(5.2%)	149(6.6%)	81(6.2%)	49(8.1%)	37(10.7%)		

*Abbreviation* *ACE* adverse childhood experience

**Table 3:** Association Between the Number of ACEs and Pain in Adulthood

	**OR (95% CI) by No. of ACEs**						***P*-value for trend**
**Pain**	**0**	**1**	**2**	**3**	**4**	** ≥ 5**	
**Model 1**^**a**^							
Headache	1[Reference]^b^	**1.193(1.012–1.406)**	**1.472(1.242–1.745)**	**1.944(1.614–2.341)**	**2.160(1.714–2.722)**	**2.999(2.295–3.918)**	*p* < 0.001
Neck pain	1[Reference]	1.152(0.947–1.401)	**1.439(1.177–1.760)**	**1.657(1.325–2.073)**	**2.127(1.627–2.782)**	**2.981(2.201–4.037)**	*p* < 0.001
Shoulder pain	1[Reference]	1.115(0.946–1.315)	**1.426(1.203–1.689)**	**1.754(1.455–2.116)**	**2.106(1.672–2.652)**	**2.840(2.172–3.714)**	*p* < 0.001
Arm pain	1[Reference]	**1.228(1.020–1.478)**	**1.664(1.378–2.009)**	**1.872(1.518–2.308)**	**2.029(1.564–2.633)**	**3.273(2.455–4.363)**	*p* < 0.001
Wrist pain	1[Reference]	**1.283(1.038–1.587)**	**1.678(1.351–2.083)**	**1.846(1.451–2.348)**	**2.091(1.558–2.808)**	**3.174(2.297–4.388)**	*p* < 0.001
Finger pain	1[Reference]	1.207(0.982–1.483)	**1.538(1.245–1.899)**	**1.619(1.276–2.054)**	**1.879(1.403–2.516)**	**3.189(2.332–4.362)**	*p* < 0.001
Chest pain	1[Reference]	**1.351(1.064–1.715)**	**1.540(1.202–1.974)**	**1.951(1.493–2.551)**	**2.525(1.843–3.460)**	**3.800(2.694–5.360)**	*p* < 0.001
Stomachache	1[Reference]	**1.300(1.067–1.586)**	**1.419(1.152–1.747)**	**2.001(1.602–2.499)**	**2.439(1.867–3.187)**	**3.387(2.503–4.582)**	*p* < 0.001
Back pain	1[Reference]	**1.265(1.054–1.518)**	**1.446(1.195–1.750)**	**1.651(1.336–2.042)**	**2.346(1.827–3.013)**	**2.508(1.855–3.391)**	*p* < 0.001
Waist pain	1[Reference]	**1.196(1.036–1.381)**	**1.558(1.343–1.807)**	**1.900(1.610–2.241)**	**2.426(1.977–2.975)**	**3.194(2.501–4.078)**	*p* < 0.001
Buttock pain	1[Reference]	1.065(0.829–1.368)	**1.570(1.224–2.014)**	**1.699(1.286–2.244)**	**1.645(1.151–2.351)**	**2.922(2.018–4.232)**	*p* < 0.001
Leg pain	1[Reference]	1.140(0.973–1.334)	**1.530(1.302–1.798)**	**1.558(1.296–1.873)**	**1.944(1.550–2.438)**	**2.853(2.198–3.704)**	*p* < 0.001
Knee pain	1[Reference]	1.093(0.934–1.280)	**1.511(1.286–1.774)**	**1.746(1.458–2.090)**	**2.006(1.603–2.510)**	**2.810(2.165–3.646)**	*p* < 0.001
Ankle pain	1[Reference]	1.174(0.941–1.465)	**1.688(1.353–2.107)**	**1.752(1.365–2.248)**	**2.531(1.898–3.376)**	**3.508(2.539–4.846)**	*p* < 0.001
Toe pain	1[Reference]	1.244(0.961–1.609)	**1.590(1.223–2.067)**	**1.483(1.095–2.008)**	**1.991(1.395–2.842)**	**2.713(1.824–4.036)**	*p* < 0.001
**Model 2**^**c**^							
Headache	1[Reference]	1.173(0.976–1.410)	**1.590(1.317–1.919)**	**2.119(1.726–2.602)**	**2.377(1.848–3.057)**	**3.194(2.391–4.265)**	*p* < 0.001
Neck pain	1[Reference]	1.170(0.940–1.456)	**1.566(1.253–1.956)**	**1.934(1.515–2.468)**	**2.429(1.818–3.245)**	**3.164(2.278–4.395)**	*p* < 0.001
Shoulder pain	1[Reference]	1.170(0.940–1.456)	**1.538(1.276–1.854)**	**1.965(1.600–2.412)**	**2.382(1.857–3.055)**	**3.004(2.248–4.014)**	*p* < 0.001
Arm pain	1[Reference]	1.218(0.989–1.501)	**1.908(1.550–2.350)**	**2.113(1.677–2.661)**	**2.418(1.829–3.196)**	**3.614(2.651–4.926)**	*p* < 0.001
Wrist pain	1[Reference]	1.228(0.967–1.559)	**1.790(1.409–2.274)**	**2.091(1.607–2.719)**	**2.344(1.707–3.218)**	**3.430(2.422–4.858)**	*p* < 0.001
Finger pain	1[Reference]	1.206(0.956–1.520)	**1.682(1.330–2.126)**	**1.861(1.433–2.416)**	**2.167(1.582–2.968)**	**3.800(2.720–5.310)**	*p* < 0.001
Chest pain	1[Reference]	**1.341(1.024–1.755)**	**1.703(1.294–2.242)**	**2.253(1.679–3.022)**	**2.971(2.118–4.167)**	**3.881(2.662–5.659)**	*p* < 0.001
Stomachache	1[Reference]	**1.313(1.053–1.637)**	**1.520(1.208–1.911)**	**2.258(1.772–2.878)**	**2.617(1.957–3.500)**	**3.565(2.573–4.939)**	*p* < 0.001
Back pain	1[Reference]	**1.242(1.013–1.523)**	**1.584(1.285–1.952)**	**1.800(1.426–2.272)**	**2.571(1.959–3.372)**	**2.555(1.842–3.545)**	*p* < 0.001
Waist pain	1[Reference]	**1.220(1.041–1.430)**	**1.716(1.457–2.019)**	**2.086(1.739–2.501)**	**2.532(2.024–3.168)**	**3.333(2.558–4.343)**	*p* < 0.001
Buttock pain	1[Reference]	1.013(0.765–1.342)	**1.727(1.314–2.270)**	**1.825(1.345–2.476)**	**1.846(1.263–2.699)**	**3.102(2.085–4.616)**	*p* < 0.001
Leg pain	1[Reference]	1.140(0.957–1.359)	**1.677(1.403–2.004)**	**1.754(1.434–2.146)**	**2.182(1.707–2.789)**	**3.132(2.364–4.150)**	*p* < 0.001
Knee pain	1[Reference]	1.020(0.856–1.216)	**1.593(1.335–1.901)**	**1.915(1.573–2.330)**	**2.150(1.686–2.741)**	**2.850(2.148–3.780)**	*p* < 0.001
Ankle pain	1[Reference]	1.054(0.824–1.350)	**1.749(1.372–2.229)**	**1.934(1.478–2.531)**	**2.683(1.968–3.659)**	**3.691(2.613–5.215)**	*p* < 0.001
Toe pain	1[Reference]	1.150(0.860–1.537)	**1.710(1.280–2.283)**	**1.695(1.222–2.353)**	**2.300(1.576–3.355)**	**3.145(2.070–4.779)**	*p* < 0.001
**Model 3**^**d**^							
Headache	1[Reference]	1.136(0.736–1.753)	**1.700(1.091–2.648)**	**2.060(1.252–3.390)**	1.514(0.784–2.923)	**2.320(1.178–4.569)**	*P* < 0.001
Neck pain	1[Reference]	0.983(0.584–1.654)	**1.868(1.125–3.103)**	**2.061(1.165–3.646)**	1.608(0.760–3.402)	**3.055(1.471–6.345)**	*P* < 0.001
Shoulder pain	1[Reference]	1.103(0.705–1.725)	**1.766(1.123–2.776)**	**2.590(1.574–4.260)**	**2.120(1.117–4.027)**	**2.367(1.181–4.744)**	*P* < 0.001
Arm pain	1[Reference]	1.232(0.751–2.022)	**1.798(1.091–2.963)**	**2.575(1.496–4.432)**	1.407(0.665–2.976)	**2.606(1.257–5.401)**	*P* = 0.001
Wrist pain	1[Reference]	1.102(0.617–1.968)	1.582(0.881–2.840)	**3.228(1.763–5.910)**	1.660(0.723–3.809)	**3.678(1.700–7.958)**	*p* < 0.001
Finger pain	1[Reference]	1.182(0.701–1.990)	1.681(0.993–2.844)	**1.917(1.061–3.462)**	1.190(0.524–2.704)	**3.704(1.805–7.600)**	*P* = 0.001
Chest pain	1[Reference]	1.632(0.819–3.251)	1.809(0.888–3.683)	**3.426(1.659–7.077)**	**3.222(1.347–7.710)**	2.485(0.919–6.718)	*P* = 0.001
Stomachache	1[Reference]	1.317(0.8022.162)	1.591(0.949–2.665)	1.495(0.815–2.744)	**3.112(1.602–6.046)**	2.039(0.939–4.429)	*P* = 0.003
Back pain	1[Reference]	1.259(0.763–2.078)	**1.933(1.167–3.202)**	**2.505(1.437–4.368)**	**2.471(1.242–4.916)**	1.690(0.750–3.806)	*P* = 0.001
Waist pain	1[Reference]	1.046(0.708–1.547)	**1.849(1.243–2.750)**	**2.243(1.428–3.524)**	**2.324(1.314–4.111)**	**2.800(1.494–5.249)**	*p* < 0.001
Buttock pain	1[Reference]	0.528(0.266–1.048)	1.209(0.650–2.250)	**2.041(1.057–3.939)**	1.301(0.517–3.277)	1.897(0.769–4.677)	*P* = 0.007
Leg pain	1[Reference]	0.915(0.607–1.379)	**1.634(1.081–2.468)**	1.536(0.946–2.491)	1.372(0.730–2.577)	**2.327(1.217–4.450)**	*P* = 0.001
Knee pain	1[Reference]	0.855(0.560–1.306)	**1.803(1.191–2.729)**	**1.958(1.221–3.141)**	1.174(0.612–2.250)	**3.305(1.752–6.235)**	*p* < 0.001
Ankle pain	1[Reference]	1.405(0.776–2.543)	**1.944(1.065–3.546)**	**2.711(1.415–5.197)**	2.217(0.974–5.048)	**3.650(1.625–8.200)**	*p* < 0.001
Toe pain	1[Reference]	1.500(0.741–3.038)	1.927(0.941–3.948)	**2.569(1.175–5.619)**	**3.821(1.581–9.236)**	**2.786(1.021–7.606)**	*P* = 0.001
**Model 4**^**e**^							
Headache	1[Reference]	1.137(0.724–1.785)	1.445(0.909–2.296)	**1.744(1.035–2.940)**	1.324(0.662–2.647)	1.738(0.851–3.550)	*P* = 0.035
Neck pain	1[Reference]	0.990(0.578–1.694)	1.646(0.971–2.791)	1.776(0.981–3.216)	1.320(0.597–2.916)	**2.466(1.147–5.304)**	*P* = 0.007
Shoulder pain	1[Reference]	1.124(0.704–1.795)	1.448(0.897–2.336)	**2.279(1.345–3.863)**	1.960(0.987–3.891)	1.816(0.865–3.812)	*P* = 0.002
Arm pain	1[Reference]	1.215(0.723–2.043)	1.471(0.866–2.497)	**2.183(1.231–3.872)**	1.195(0.539–2.648)	1.904(0.878–4.128)	*P* = 0.030
Wrist pain	1[Reference]	1.149(0.629–2.098)	1.346(0.729–2.484)	**2.889(1.534–5.440)**	1.459(0.606–3.513)	**2.840(1.261–6.399)**	*P* = 0.001
Finger pain	1[Reference]	1.160(0.676–1.992)	1.426(0.823–2.471)	1.585(0.855–2.937)	0.996(0.422–2.350)	**3.069(1.433–6.570)**	*P* = 0.019
Chest pain	1[Reference]	1.635(0.797–3.356)	1.456(0.688–3.082)	**2.839(1.328–6.068)**	**2.778(1.107–6.971)**	1.609(0.551–4.693)	*P* = 0.029
Stomachache	1[Reference]	1.456(0.852–2.489)	1.283(0.732–2.249)	1.114(0.574–2.159)	**2.708(1.297–5.655)**	1.437(0.611–3.380)	*P* = 0.138
Back pain	1[Reference]	1.231(0.730–2.074)	1.614(0.950–2.744)	**2.094(1.165–3.763)**	**2.148(1.031–4.475)**	1.313(0.557–3.098)	*P* = 0.024
Waist pain	1[Reference]	1.087(0.725–1.630)	**1.601(1.057–2.425)**	**2.033(1.267–3.261)**	**2.264(1.245–4.117)**	**2.276(1.169–4.432)**	*p* < 0.001
Buttock pain	1[Reference]	0.484(0.238–0.984)	0.969(0.503–1.864)	1.665(0.836–3.315)	1.171(0.446–3.079)	1.283(0.495–3.326)	*p* = 0.001
Leg pain	1[Reference]	0.907(0.590–1.396)	1.368(0.884–2.118)	1.250(0.747–2.092)	1.254(0.641–2.453)	1.785(0.889–3.582)	*P* = 0.069
Knee pain	1[Reference]	0.851(0.548–1.324)	1.534(0.991–2.374)	**1.706(1.037–2.805)**	1.077(0.541–2.146)	**2.562(1.304–5.035)**	*P* = 0.048
Ankle pain	1[Reference]	1.439(0.771–2.687)	1.662(0.877–3.150)	**2.376(1.193–4.734)**	2.109(0.873–5.091)	**2.842(1.192–6.776)**	*p* = 0.004
Toe pain	1[Reference]	1.548(0.746–3.216)	1.807(0.856–3.813)	**2.433(1.077–5.500)**	**4.411(1.740–11.179)**	2.068(0.704–6.076)	*p* = 0.006

*Abbreviations,* *ACE* Adverse Childhood Experience, *OR* Odds Ratio

^*a*^ Model 1 was the crude model

^*b*^ Reference: No ACE exposure

^*c*^ Model 2 was adjusted for age, sex, body mass index

^*d*^ Model 3 was adjusted for age, sex, body mass index, area of residence, educational level, childhood family economic hardship, smoking and drinking status, marital status, physical activity

^*e*^ Model 4 was adjusted for age, sex, body mass index, area of residence, educational level, childhood family economic hardship, smoking and drinking status, marital status, physical activity and 14 chronic diseases (Hypertension, dyslipidemia, diabetes, heart disease, stroke, chronic lung disease, asthma, liver disease, cancer, digestive disease, kidney disease, arthritis, psychiatric disease, and memory-related disease)

**Fig. 2:**
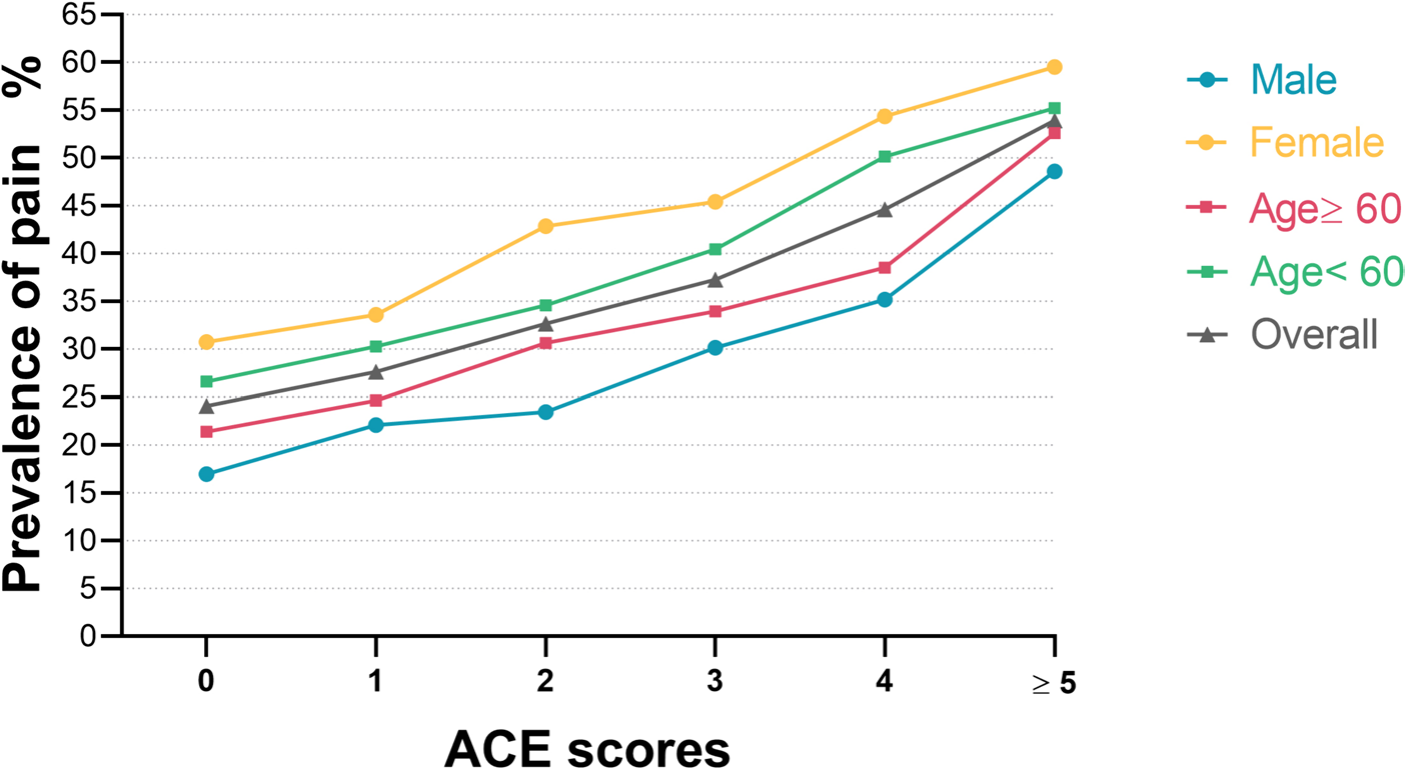
Prevalence of pain by Number of ACE in the Overall Study Population Stratified by Age and Sex

Table 4 presents the associations of ACE groups with body pains (single pain and multiple pain) in the overall study population and subgroups. The individuals who were exposed to 5 or more ACEs had an approximately threefold risk compared with those who did not experience ACEs (OR, 2.713; 95% CI, 2.110–3.487). Consistent outcomes were found in the subgroups, with significant dose–response associations (Table 4). Similar results were found in the associations between ACEs with multiple and single pain (Table S3 and S4) and no covariates significantly modified the association between ACEs with multiple and single pain excluding the area of residence and childhood economic hardship. In addition, stratified analyses showed that higher cumulative ACE scores were linked to a heightened prevalence of pain in the overall population and subgroups (Fig. 2).

**Table 4:** Association Between the Number of ACEs and pain in the Overall Study Population and Subgroups

	**OR (95% CI) by No. of ACEs**						***P*-value for trend**	***P*-value for interaction**
**Characteristic**^**a**^	**0**	**1**	**2**	**3**	**4**	** ≥ 5**		
**Overall study population**	1 [Reference]^b^	**1.156(1.016–1.317)**	**1.335(1.163–1.532)**	**1.591(1.360–1.862)**	**2.014(1.648–2.461)**	**2.713(2.110–3.487)**	***p* < 0.001**	
**Subgroups**								
** Sex**								
Male	1 [Reference]	**1.289(1.054–1.577)**	**1.311(1.060–1.622)**	**1.760(1.392–2.225)**	**2.113(1.572–2.841)**	**3.277(2.297–4.674)**	***p* < 0.001**	0.445
Female	1 [Reference]	1.072(0.903–1.273)	**1.387(1.154–1.666)**	**1.465(1.181–1.816)**	**1.949(1.478–2.570)**	**2.317(1.621–3.311)**	***p* < 0.001**	
**Age**								
< 60	1 [Reference]	1.165(0.952–1.426)	**1.430(1.156–1.770)**	**1.708(1.340–2.177)**	**2.105(1.541–2.875)**	**3.000(2.073–4.341)**	***p* < 0.001**	0.162
≥ 60	1 [Reference]	1.145(0.965–1.357)	**1.271(1.059–1.524)**	**1.528(1.241–1.882)**	**1.984(1.524–2.583)**	**2.502(1.771–3.533)**	***p*** **< 0.001**	
**Childhood family economic hardship**								
No	1 [Reference]	1.124(0.957–1.321)	**1.324(1.109–1.580)**	**1.728(1.397–2.138)**	**2.180(1.630–2.915)**	**3.801(2.534–5.700)**	***p* < 0.001**	0.085
Yes	1 [Reference]	1.194(0.956–1.491)	**1.345(1.074–1.685)**	**1.462(1.147–1.863)**	**1.870(1.400–2.496)**	**2.239(1.603–3.129)**	***p* < 0.001**	
**Area of residence**								
Rural	1 [Reference]	1.141(0.986–1.320)	**1.305(1.118–1.524)**	**1.586(1.330–1.892)**	**1.964(1.569–2.459)**	**2.537(1.909–3.370)**	***p* < 0.001**	0.511
Urban	1 [Reference]	1.200(0.897–1.606)	**1.494(1.098–2.032)**	**1.667(1.165–2.384)**	**2.361(1.502–3.710)**	**3.490(2.023–6.021)**	***p* < 0.001**	
**Educational level completed**								
None	1 [Reference]	1.007(0.767–1.323)	**1.389(1.042–1.851)**	**1.367(0.980–1.906)**	**2.483(1.616–3.814)**	**2.083(1.198–3.624)**	***p* < 0.001**	0.743
Home School to Primary School	1 [Reference]	1.217(0.994–1.492)	**1.284(1.035–1.593)**	**1.634(1.288–2.072)**	**1.610(1.188–2.183)**	**2.748(1.907–3.960)**	***p* < 0.001**	
Middle school or above	1 [Reference]	1.174(0.942–1.462)	**1.383(1.094–1.749)**	**1.741(1.321–2.296)**	**2.409(1.696–3.422)**	**3.141(2.006–4.917)**	***p* < 0.001**	
**Physical Activities**								
No activity	1 [Reference]	1.323(0.886–1.977)	**1.879(1.236–2.858)**	**1.755(1.078–2.855)**	**2.961(1.492–5.877)**	**3.549(1.479–8.516)**	***p* < 0.001**	0.251
With activity	1 [Reference]	1.135(0.989–1.302)	**1.281(1.107–1.484)**	**1.575(1.333–1.860)**	**1.932(1.565–2.386)**	**2.600(1.997–3.385)**	***p* < 0.001**	

*Abbreviations,* *ACE* Adverse Childhood Experience, *OR* Odds Ratio

^*a*^The model was adjusted was adjusted for age, sex, body mass index, area of residence, educational level, childhood family economic hardship, smoking and drinking status, marital status, physical activity, except for the stratified variables in each subgroup

^*b*^Reference, No *ACE* exposure

**Fig. 3:**
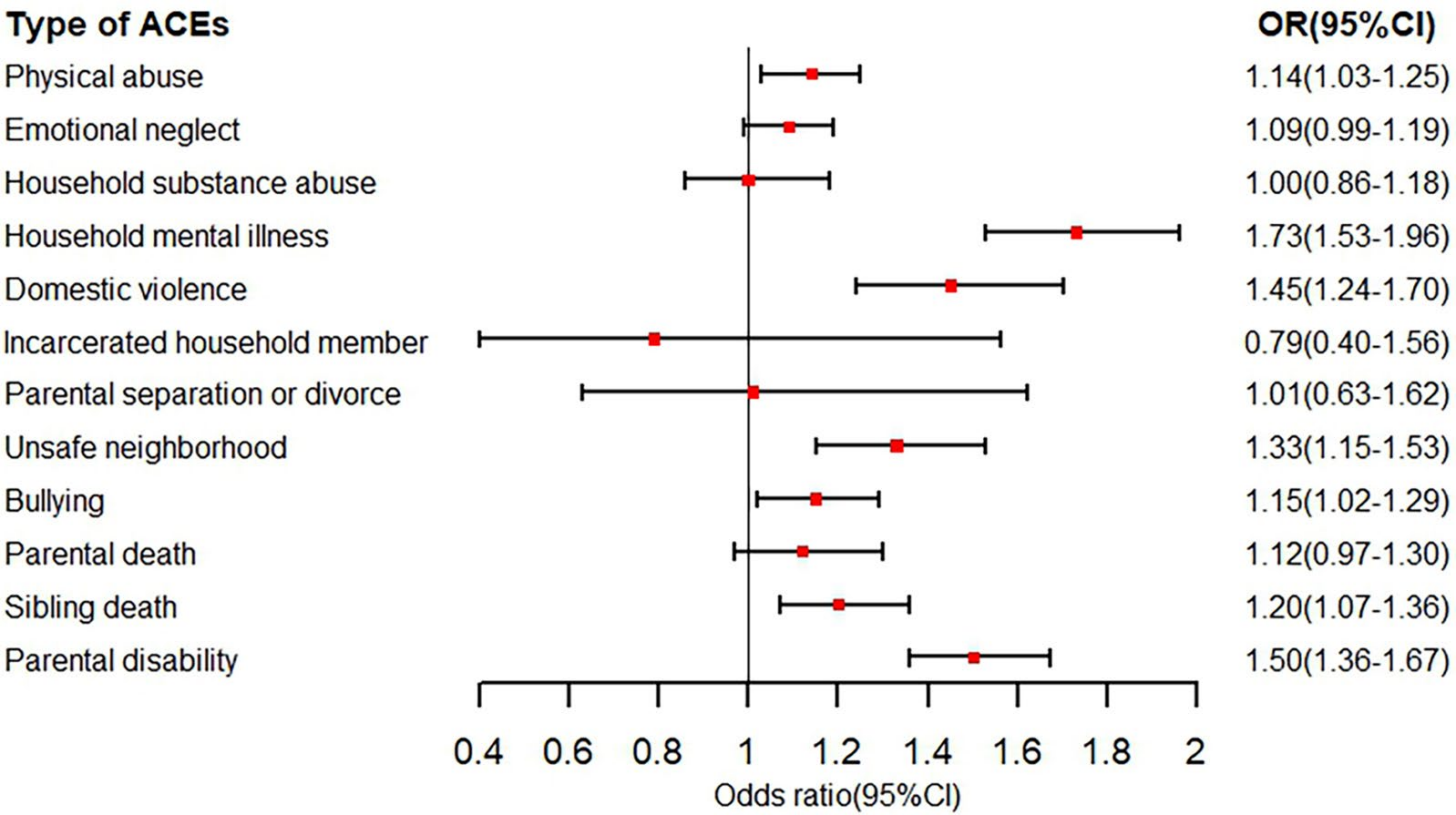
Associations Between Each ACE Indicator and Pain. Model was adjusted for age, sex, body mass index, area of residence, educational level, childhood family economic hardship, smoking and drinking status, marital status, physical activity and 14 chronic diseases (hypertension, dyslipidemia, diabetes, heart disease, stroke, chronic lung disease, asthma, liver disease, cancer, digestive disease, kidney disease, arthritis, psychiatric disease, and memory-related disease)

The associations of each ACE indicator with pain was analyzed (Fig. 3 and Figure S1–S2). Among the 12 ACE indicators, 11 were associated with higher risk of having single pain, and 7 indicators were statistically significant (including physical abuse, household mental illness, domestic violence, unsafe neighborhood, bullying, sibling death and parental disability) (Fig. 3). Similar results were found in the results of the associations between each ACE indicator and multiple pain (Figure S2). In the associations of each body pain with each ACE indicator, most ACE indicators (except incarcerated household members) were associated with increased risk of each body pain. In addition, these ACE indicators were associated with increased risk of having waist pain, and waist pain exhibited the most significant association with ACE indicators among 15 body pains (Figure S3–S17).

## Discussion

This study examined the cross-sectional associations between ACEs and self-reported pain in middle-aged and elderly adults in China. The findings also suggest significant dose–response associations between the number of ACEs to which individuals were exposed and the prevalence of pain. Furthermore, physical activity and sociodemographic and socioeconomic characteristics did not significantly modify the primary associations.

The associations of headache or related few body pains with ACEs has been previously demonstrated in adolescent and adults, and the results of the present study are consistent with those of earlier research [30]. However, the exact mechanism underlying the association is unclear. Various mechanisms may be possible, and many studies over the past few years focused on psychological factors. ACEs increase a range of mental burdens, including depression, anxiety, posttraumatic stress disorder, and psychiatric pharmacotherapy, especially in those who have experienced more ACEs [1, 31–34]. Mental health problems are strongly associated with the increased odds of having pain [35]. One stress–diatheses model suggests that ACEs can predict threat appraisal and posttraumatic distress, which can further predict pain [36]. Posttraumatic distress was more prevalent among adolescents with chronic pain than those without pain, and the effects of pain interventions worsened over time [37, 38]. Pain interventions are usually psychiatric pharmacotherapy, and ACEs have been demonstrated to possibly increase the risk to opioid use disorder among individuals with chronic pain who are undergoing prolonged opioid analgesic therapy [34]. Another explanation for ACEs with psychological factors is that ACEs may cause hyperalgesia through emotions, and that this nociceptive hypersensitivity has the ability to emotionally modulate pain [39].

ACEs have been shown to exert broad-ranging effects on the neural, endocrine, immune, and metabolic physiological systems [40], which, in turn, increase the risk of many chronic diseases [10]. This finding is consistent with the findings of the current study, and many of the complications of these chronic diseases are associated with pain [16]. One explanation of these associations is ACEs changing the brain structure and function [41–43]. ACEs were found to decrease prefrontal cortex gray matter volumes and hippocampal volumes in adolescents, adults, and animal studies [42–44], and decreased or increased amygdala volumes [45]. The aforementioned change in central stress-regulating structures can increase the risk of multisystem chronic diseases[46]. ACEs significantly affect stress responses, which are regulated by the hypothalamic–pituitary–adrenal and autonomic (sympathetic/parasympathetic) nervous system pathways. These pathways are centrally regulated by corticolimbic structures, which encompass the prefrontal cortex, hippocampus, and amygdala [12], A meta-analysis showed that in task-based functional MRI studies, patients with chronic pain or healthy subjects, compared to those who have not experienced ACEs, those who have experienced ACEs have received one significant cluster in the limbic and parental lobe, specifically: left posterior cingulate gyrus, inferior parietal lobule and left precuneus. And in structural MRI experiments, chronic pain individuals exposed to ACEs compared with chronic pain participants not exposed to ACEs: no studies were identified because of the small group size [47].

Another possible explanation for ACEs increasing pain risk is that they significantly increase unhealthy behavior [22], such as alcohol consumption, smoking and risky sexual behavior, which persist into adulthood [1], because the behavior established in developing neural networks has a lasting effect [48]. ACE exposure resulted in a fivefold increase in the risk of initiating smoking early and an almost threefold surge in the risk of engaging in heavy smoking [49], and a recent study indicated a significant association between smoking and pain [50]. The risk of drinking was also found to be increased in a previous study [51]. The present study similarly found that ACEs increased the risk of drinking.

In the associations of each body pain with each ACE indicator, most ACE indicators (except incarcerated household members) were associated with increased pain. And the only inconsistent results may be related to its low prevalence, because this indicator was the lowest prevalence of all ACE indicators in the total population and among patients with pain (single pain: 0.05%, multiple pain: 0.07%). In addition, the highest number of significant outcomes with ACE indicators is waist pain among all body pains (7 ACE indicators were statistically significant). The reason may be that waist pain covers a spectrum of different types of pain, including nociceptive pain, neuropathic pain, and nociplastic pain [52]. It also had the highest prevalence among all ACE groups in the present study.

One main focus of this study was to explore whether physical activity and demographic characteristics modified the associations between ACEs and pain. The results showed that age, sex, educational level, area of residence, childhood economic hardship, and physical activity did not significantly alter these associations in the subgroup analyses, similar to the results of previous studies [10, 53] Conversely, a recent study found that sociodemographic characteristics may weaken the associations of ACEs with the onset of dementia [9]. In addition, after physical activities were categorized into vigorous, moderate, and low physical activities, vigorous and low physical activities were found to be significantly associated with cumulative ACE scores. The trend analysis showed the same result, suggesting a subsequent need to re-explore whether physical activity modifies this relationship in large samples with high cumulative ACEs scores.

### Strengths and Limitations

This study has significant strengths, firstly, the large study sample used to explore the associations between ACEs and 15 different body pains. Secondly, it considered the pain sites, prevalence, and potential modifying factors. However, it has several limitations. Firstly, a substantial number of participants were excluded from the primary analyses due to either loss to follow-up or missing data. This exclusion could have potentially introduced selection bias and limited the generalizability of the study results. Moreover, the participants who were excluded may have been exposed to a higher frequency of pain, potentially resulting in an underestimation of pain prevalence. Given that pain was made up of 15 body pains, these pains may have occurred as a direct result of some musculoskeletal disease or other chronic disease, because many of the participants were suffering from one or more of these diseases (Table 1). However, ACEs have been shown to increase odds in many chronic diseases [10]. The outcomes derived from the imputed datasets were in agreement with those from complete-case analyses, indicating the reliability of the study results. Secondly, ACEs were assessed via retrospective assessment, which may have potentially introduced memory-related biases. A previous investigation consistently demonstrated that when it comes to ACEs, retrospective assessments exhibit strong test–retest reliability [54], and cannot be easily substituted with prospective measures [55]. Thirdly, no relevant frequency, severity, and persistence of pain and ACEs are available in the CHARLS data, and this limited may have affected the main findings of this study. The operationalization of ACEs and pain has been shown to have dose–response associations with poor health outcomes [56, 57] Fourthly, pain and ACEs include a wide range of indicators. However, some reported and widely used ACE indicators have caused considerable suffering. These ACE indicators [4, 22] were not included due to lack of relevant data. Fifthly, the self-reported nature of pain may differ from the use of diagnostic pain (e.g. the international statistical classification of diseases and related health problems 10th revision), which may lead to different findings. Finally, some relevant variables that may modify the study associations were not included in the model, such as sleep-related problems [58], and specific chronic diseases related to memory, such as dementia [59].

## Conclusions

In this large nationwide-representative population in China, dose–response associations were observed between cumulative exposure to ACEs and self-reported pain odds. However, no significant modification was found for the associations by physical activity and demographic and socioeconomic factors. The results indicate that ACEs result in high burden and serious and long-lasting consequences. Thus, a subsequent study is needed to obtain an effective strategy that reduces the risk of pain associated with ACEs.

### Supplementary Information

Supplementary Material 1: Table S1. Questionnaire Items and Prevalence of Each ACE Indicator. Table S2. Associations Between the Number of ACEs and Body Pains, With Imputed Data Sets. Table S3. Associations Between the Number of ACEs and Single Pain in the Overall Study Population and Subgroups. Table S4. Associations Between the Number of ACEs and Multiple Pain in the Overall Study Population and Subgroups. Figure S1. Associations Between Individual ACE Indicator and Single pain. Figure S2. Associations Between Individual ACE Indicator and multiple pain. Figure S3. Associations Between Individual ACE Indicator and Headache. Figure S4. Associations Between Individual ACE Indicator and Neck Pain. Figure S5. Associations Between Individual ACE Indicator and Shoulder Pain. Figure S6. Associations Between Individual ACE Indicator and Arm Pain. Figure S7. Associations Between Individual ACE Indicator and Wrist Pain. Figure S8. Associations Between Individual ACE Indicator and Finger Pain. Figure S9. Associations Between Individual ACE Indicator and Chest Pain. Figure S10. Associations Between Individual ACE Indicator and Stomachache. Figure S11. Associations Between Individual ACE Indicator and Back Pain. Figure S12. Associations Between Individual ACE Indicator and Waist Pain. Figure S13. Associations Between Individual ACE Indicator and Bottock Pain. Figure S14. Associations Between Individual ACE Indicator and Leg Pain. Figure S15. Associations Between Individual ACE Indicator and Knee Pain. Figure S16. Associations Between Individual ACE Indicator and Ankle Pain. Figure S17. Associations Between Individual ACE Indicator and Toe Pain.

## Data Availability

The data that support the findings of this study are available from CHARLS project site, subject to registration and application process. Further details can be found at https://charls.charlsdata.com/pages/Data/.

## Abbreviations

ACEs: Adverse childhood experiences
CHARLS: The China Health and Retirement Longitudinal Study
STROBE: The Strengthening the Reporting of Observational Studies in Epidemiology
BMI: Body mass index; ORs: odds ratios
