# Supplementary material for: Associations between adverse childhood experiences and pain in middle-aged and older adults: findings from the China Health and Retirement Longitudinal Study
Source: BMC Public Health. 2024 Jul 2;24:1760. doi: 10.1186/s12889-024-19239-6 (PMC11218369; doi:10.1186/s12889-024-19239-6)
Supplement: Supplementary file 1 — Supplementary Material 1: Table S1. Questionnaire Items and Prevalence of Each ACE Indicator. Table S2. Associations Between the Number of ACEs and Body Pains, With Imputed Data Sets. Table S3. Associations Between the Number of ACEs and Single Pain in the Overall Study Population and Subgroups. Table S4. Associations Between the Number of ACEs and Multiple Pain in the Overall Study Population and Subgroups. Figure S1. Associations Between Individual ACE Indicator and Single pain. Figure S2. Associations Between Individual ACE Indicator and multiple pain. Figure S3. Associations Between Individual ACE Indicator and Headache. Figure S4. Associations Between Individual ACE Indicator and Neck Pain. Figure S5. Associations Between Individual ACE Indicator and Shoulder Pain. Figure S6. Associations Between Individual ACE Indicator and Arm Pain. Figure S7. Associations Between Individual ACE Indicator and Wrist Pain. Figure S8. Associations Between Individual ACE Indicator and Finger Pain. Figure S9. Associations Between Individual ACE Indicator and Chest Pain. Figure S10. Associations Between Individual ACE Indicator and Stomachache. Figure S11. Associations Between Individual ACE Indicator and Back Pain. Figure S12. Associations Between Individual ACE Indicator and Waist Pain. Figure S13. Associations Between Individual ACE Indicator and Bottock Pain. Figure S14. Associations Between Individual ACE Indicator and Leg Pain. Figure S15. Associations Between Individual ACE Indicator and Knee Pain. Figure S16. Associations Between Individual ACE Indicator and Ankle Pain. Figure S17. Associations Between Individual ACE Indicator and Toe Pain. [file 12889_2024_19239_MOESM1_ESM.docx]

**Supplementary Online Content**

**Supplementary Covariates**

Marital status was categorized into currently married/cohabitated and unmarried. The latter included never married, divorced, and widowed. Educational level completed was divided into three groups, i.e., none, home school to primary school and middle school above. Childhood economic hardship was ascertained by the question “When you were a child before the age of 17 years, compared to the average family in the same community/village at that time, how was your family’s financial situation?” If the participant chose “a lot worse” or “somewhat worse”, he/she was encoded as having economic hardship during childhood. In contrast, if “a lot better”, “somewhat better” or “same as them” was selected, the participants were grouped as without economic hardship during their childhood. Smoking status were categorized into never, former and current users based on self-report. And drink status were categorized into never, ≤1/month and ＞1/month. BMI of 28 or greater were defined as having obesity based on the recommended standard for Chinese adults(1).

The definition of chronic diseases have reported by previous study(2) and the 14 chronic diseases were dyslipidemia, heart disease, stroke, chronic lung disease, asthma, liver disease, cancer, digestive disease, kidney disease, arthritis, psychiatric disease, and memory-related disease (including Alzheimer’s disease, Parkinson’s disease, and cerebral atrophy) were defined based on self-report of a physician’s diagnosis.

Physical activity was ascertained by the question “Now we would like to ask about the amount of time you spend on different types of physical activities in a usual week?” There are three options for physical activity (vigorous physical activities, moderate physical activities and low physical activities) and three types of frequency and duration related to these physical activities.

STROBE Statement—checklist of items that should be included in reports of observational studies

|  | **Item No** | **Recommendation** | **Page  No** |
| --- | --- | --- | --- |
| **Title and abstract** | 1 | (*a*) Indicate the study’s design with a commonly used term in the title or the abstract | 1 |
|  |  | (*b*) Provide in the abstract an informative and balanced summary of what was done and what was found | 1 |
| **Introduction** | | | |
| Background/rationale | 2 | Explain the scientific background and rationale for the investigation being reported | 2-3 |
| Objectives | 3 | State specific objectives, including any prespecified hypotheses | 2-3 |
| **Methods** | | | |
| Study design | 4 | Present key elements of study design early in the paper | 3 |
| Setting | 5 | Describe the setting, locations, and relevant dates, including periods of recruitment, exposure, follow-up, and data collection | 3-5 |
| Participants | 6 | (*a*) *Cohort study*—Give the eligibility criteria, and the sources and methods of selection of participants. Describe methods of follow-up  *Case-control study*—Give the eligibility criteria, and the sources and methods of case ascertainment and control selection. Give the rationale for the choice of cases and controls  *Cross-sectional study*—Give the eligibility criteria, and the sources and methods of selection of participants |  |
|  |  | (*b*) *Cohort study*—For matched studies, give matching criteria and number of exposed and unexposed  *Case-control study*—For matched studies, give matching criteria and the number of controls per case | 3 |
| Variables | 7 | Clearly define all outcomes, exposures, predictors, potential confounders, and effect modifiers. Give diagnostic criteria, if applicable | 3-5 |
| Data sources/ measurement | 8* | For each variable of interest, give sources of data and details of methods of assessment (measurement). Describe comparability of assessment methods if there is more than one group | 3-5 |
| Bias | 9 | Describe any efforts to address potential sources of bias |  |
| Study size | 10 | Explain how the study size was arrived at | 3 |
| Quantitative variables | 11 | Explain how quantitative variables were handled in the analyses. If applicable, describe which groupings were chosen and why | 3-4 |
| Statistical methods | 12 | (*a*) Describe all statistical methods, including those used to control for confounding | 5-6 |
|  |  | (*b*) Describe any methods used to examine subgroups and interactions | 5-6 |
|  |  | (*c*) Explain how missing data were addressed |  |
|  |  | (*d*) *Cohort study*—If applicable, explain how loss to follow-up was addressed  *Case-control study*—If applicable, explain how matching of cases and controls was addressed  *Cross-sectional study*—If applicable, describe analytical methods taking account of sampling strategy |  |
|  |  | (*e*) Describe any sensitivity analyses |  |

Continued on next page

| **Results** | | | |
| --- | --- | --- | --- |
| Participants | 13* | (a) Report numbers of individuals at each stage of study—eg numbers potentially eligible, examined for eligibility, confirmed eligible, included in the study, completing follow-up, and analysed | 6 |
|  |  | (b) Give reasons for non-participation at each stage | 3 |
|  |  | (c) Consider use of a flow diagram | 4 |
| Descriptive data | 14* | (a) Give characteristics of study participants (eg demographic, clinical, social) and information on exposures and potential confounders | Table 1 |
|  |  | (b) Indicate number of participants with missing data for each variable of interest | Figure 1 |
|  |  | (c) *Cohort study*—Summarise follow-up time (eg, average and total amount) |  |
| Outcome data | 15* | *Cohort study*—Report numbers of outcome events or summary measures over time |  |
|  |  | *Case-control study—*Report numbers in each exposure category, or summary measures of exposure |  |
|  |  | *Cross-sectional study—*Report numbers of outcome events or summary measures | Figure1 |
| Main results | 16 | (*a*) Give unadjusted estimates and, if applicable, confounder-adjusted estimates and their precision (eg, 95% confidence interval). Make clear which confounders were adjusted for and why they were included | Table 3 |
|  |  | (*b*) Report category boundaries when continuous variables were categorized |  |
|  |  | (*c*) If relevant, consider translating estimates of relative risk into absolute risk for a meaningful time period |  |
| Other analyses | 17 | Report other analyses done—eg analyses of subgroups and interactions, and sensitivity analyses | Figure 2 and Figure 3 |
| **Discussion** | | | |
| Key results | 18 | Summarise key results with reference to study objectives | 7 |
| Limitations | 19 | Discuss limitations of the study, taking into account sources of potential bias or imprecision. Discuss both direction and magnitude of any potential bias | 10 |
| Interpretation | 20 | Give a cautious overall interpretation of results considering objectives, limitations, multiplicity of analyses, results from similar studies, and other relevant evidence | 11 |
| Generalisability | 21 | Discuss the generalisability (external validity) of the study results | 7-11 |
| **Other information** | | | |
| Funding | 22 | Give the source of funding and the role of the funders for the present study and, if applicable, for the original study on which the present article is based | Title page |

*Give information separately for cases and controls in case-control studies and, if applicable, for exposed and unexposed groups in cohort and cross-sectional studies.

Note: An Explanation and Elaboration article discusses each checklist item and gives methodological background and published examples of transparent reporting. The STROBE checklist is best used in conjunction with this article (freely available on the Web sites of PLoS Medicine at http://www.plosmedicine.org/, Annals of Internal Medicine at http://www.annals.org/, and Epidemiology at http://www.epidem.com/). Information on the STROBE Initiative is available at www.strobe-statement.org.

**Table S1.** Questionnaire Items and Prevalence of Each ACE Indicator

**Table S2.** Associations Between the Number of ACEs and Body Pains, With Imputed Data Sets.

**Table S3.** Associations Between the Number of ACEs and Single Pain in the Overall Study Population and Subgroups

**Table S4.** Associations Between the Number of ACEs and Multiple Pain in the Overall Study Population and Subgroups

**Figure S1.** Associations Between Individual ACE Indicator and Single pain.

**Figure S2.** Associations Between Individual ACE Indicator and multiple pain.

**Figure S3.** Associations Between Individual ACE Indicator and Headache

**Figure S4.** Associations Between Individual ACE Indicator and Neck Pain

**Figure S5.** Associations Between Individual ACE Indicator and Shoulder Pain

**Figure S6.** Associations Between Individual ACE Indicator and Arm Pain

**Figure S7.** Associations Between Individual ACE Indicator and Wrist Pain

**Figure S8.** Associations Between Individual ACE Indicator and Finger Pain

**Figure S9.** Associations Between Individual ACE Indicator and Chest Pain

**Figure S10.** Associations Between Individual ACE Indicator and Stomachache

**Figure S11.** Associations Between Individual ACE Indicator and Back Pain

**Figure S12.** Associations Between Individual ACE Indicator and Waist Pain

**Figure S13.** Associations Between Individual ACE Indicator and Bottock Pain

**Figure 14.** Associations Between Individual ACE Indicator and Leg Pain

**Figure 15.** Associations Between Individual ACE Indicator and Knee Pain

**Figure 16.** Associations Between Individual ACE Indicator and Ankle Pain

**Figure 17.** Associations Between Individual ACE Indicator and Toe Pain

**Table S1 Questionnaire Items and Prevalence of Each ACE Indicator**

| ACE indicators | Questionnaire Items | Total Population (N= 9923) | Single Pain (N= 450) | Multiple Pain (N= 2645) |
| --- | --- | --- | --- | --- |
| Parental separation or divorce | Were your biological parents divorced (including long separation due to emotional problems) before you were 17 years? (yes^a^ or no) | 0.55% | **0.04%** | 0.23% |
| Physical abuse | When you were growing up, did your female/male guardian ever hit you? (often^a^, sometimes ^a^, rarely, or never) | 18.61% | 1.35% | 8.76% |
| Domestic violence | Have your father/mother ever beat up your mother/father? (often^a^, sometimes^a^, not very often, or never) | 4.28% | 0.40% | 2.83% |
| Emotional Neglect | 1)How much love and affection did your female guardian give you while you were growing up? (often, sometimes, rarely^a^, or never^a^) | 21.37% | 1.41% | 9.08% |
|  | 2)How much effort did your female guardian put into watching over you? (a lot, some, a little^a^, or not at all^a^) |  |  |  |
| Household mental illness | 1)Did your female/male guardian have abnormality of mind when you were young? (yes^a^ or no) | 6.94% | 0.73% | 5.22% |
|  | 2)During the years you were growing up, had your female/male guardian often showed continued signs of sadness or depression? (during all^a^, most^a^, some, or only a little of the childhood) |  |  |  |
| Household substance abuse | During the years you were growing up, did your female/male guardian ever have alcoholism or drug? (yes^a^ or no) | 5.08% | 0.41% | 2.16% |
| Incarcerated household member | During the years you were growing up, have your female/male guardian ever been arrested or sent to prison? (yes^a^ or no) | 0.28% | **0.05%** | **0.07%** |
| Unsafe neighborhood | Was it safe being out alone at night in the neighborhood where you lived as a child? (very safe,  somewhat safe, not very safe^a^, or not safe at all^a^) | 5.33% | 0.48% | 3.10% |
| Bullying | 1)When you were a child, how often were you picked on or bullied by kids in your neighborhood? (often^a^, sometimes^a^, not very often, or never) | 10.29% | 0.86% | 5.15% |
|  | 2)When you were a child, how often were you picked on or bullied by kids in your school? (often^a^, sometimes^a^, not very often, or never) |  |  |  |
| Parental death^b^ | Either of the parents was dead before participant was 17 years. (yes^a^ or no) | 8.97% | 0.41% | 2.71% |
| Parental disability | 1)Did your female/male guardian have a long time being sick on bed when you were young? (yes^a^ or no) | 12.55% | 1.09% | 7.67% |
|  | 2)Did your female/male guardian have a serious deformity when you were young? (yes^a^ or no) |  |  |  |
| Sibling death^b^ | Any of the siblings was dead before participant was 17 years. (yes^a^ or no) | 9.18% | 0.66% | 4.52% |

**Table S2 Associations Between the Number of ACEs and Body Pains, With Imputed Data Sets.**

|  | **OR (95% CI) by No. of ACEs** | | | | | | **P-value for trend** |
| --- | --- | --- | --- | --- | --- | --- | --- |
| **Pain** | **0** | **1** | **2** | **3** | **4** | **≥5** |  |
| **Model 1^a^** |  |  |  |  |  |  |  |
| Headache | 1[Reference]^b^ | **1.193(1.012-1.406)** | **1.472(1.242-1.745)** | **1.944(1.614-2.341)** | **2.160(1.714-2.722)** | **2.999(2.295-3.918)** | **p＜0.001** |
| Neck pain | 1[Reference] | 1.152(0.947-1.401) | **1.439(1.177-1.760)** | **1.657(1.325-2.073)** | **2.127(1.627-2.782)** | **2.981(2.201-4.037)** | **p＜0.001** |
| Shoulder pain | 1[Reference] | 1.115(0.946-1.315) | **1.426(1.203-1.689)** | **1.754(1.455-2.116)** | **2.106(1.672-2.652)** | **2.840(2.172-3.714)** | **p＜0.001** |
| Arm pain | 1[Reference] | **1.228(1.020-1.478)** | **1.664(1.378-2.009)** | **1.872(1.518-2.308)** | **2.029(1.564-2.633)** | **3.273(2.455-4.363)** | **p＜0.001** |
| Wrist pain | 1[Reference] | **1.283(1.038-1.587)** | **1.678(1.351-2.083)** | **1.846(1.451-2.348)** | **2.091(1.558-2.808)** | **3.174(2.297-4.388)** | **p＜0.001** |
| Finger pain | 1[Reference] | 1.207(0.982-1.483) | **1.538(1.245-1.899)** | **1.619(1.276-2.054)** | **1.879(1.403-2.516)** | **3.189(2.332-4.362)** | **p＜0.001** |
| Chest pain | 1[Reference] | **1.351(1.064-1.715)** | **1.540(1.202-1.974)** | **1.951(1.493-2.551)** | **2.525(1.843-3.460)** | **3.800(2.694-5.360)** | **p＜0.001** |
| Stomachache | 1[Reference] | **1.300(1.067-1.586)** | **1.419(1.152-1.747)** | **2.001(1.602-2.499)** | **2.439(1.867-3.187)** | **3.387(2.503-4.582)** | **p＜0.001** |
| Back pain | 1[Reference] | **1.265(1.054-1.518)** | **1.446(1.195-1.750)** | **1.651(1.336-2.042)** | **2.346(1.827-3.013)** | **2.508(1.855-3.391)** | **p＜0.001** |
| Waist pain | 1[Reference] | **1.196(1.036-1.381)** | **1.558(1.343-1.807)** | **1.900(1.610-2.241)** | **2.426(1.977-2.975)** | **3.194(2.501-4.078)** | **p＜0.001** |
| Buttock pain | 1[Reference] | 1.065(0.829-1.368) | **1.570(1.224-2.014)** | **1.699(1.286-2.244)** | **1.645(1.151-2.351)** | **2.922(2.018-4.232)** | **p＜0.001** |
| Leg pain | 1[Reference] | 1.140(0.973-1.334) | **1.530(1.302-1.798)** | **1.558(1.296-1.873)** | **1.944(1.550-2.438)** | **2.853(2.198-3.704)** | **p＜0.001** |
| Knee pain | 1[Reference] | 1.093(0.934-1.280) | **1.511(1.286-1.774)** | **1.746(1.458-2.090)** | **2.006(1.603-2.510)** | **2.810(2.165-3.646)** | **p＜0.001** |
| Ankle pain | 1[Reference] | 1.174(0.941-1.465) | **1.688(1.353-2.107)** | **1.752(1.365-2.248)** | **2.531(1.898-3.376)** | **3.508(2.539-4.846)** | **p＜0.001** |
| Toe pain | 1[Reference] | 1.244(0.961-1.609) | **1.590(1.223-2.067)** | **1.483(1.095-2.008)** | **1.991(1.395-2.842)** | **2.713(1.824-4.036)** | **p＜0.001** |
| **Model 2^c^** |  |  |  |  |  |  |  |
| Headache | 1[Reference] | **1.229(1.041-1.451)** | **1.536(1.293-1.824)** | **2.060(1.706-2.487)** | **2.240(1.772-2.832)** | **3.172(2.417-4.164)** | **p＜0.001** |
| Neck pain | 1[Reference] | 1.162(0.953-1.417) | **1.418(1.155-1.741)** | **1.594(1.267-2.005)** | **1.963(1.490-2.586)** | **2.680(1.958-3.669)** | **p＜0.001** |
| Shoulder pain | 1[Reference] | 1.146(0.971-1.353) | **1.484(1.251-1.762)** | **1.853(1.533-2.239)** | **2.175(1.723-2.747)** | **2.995(2.281-3.932)** | **p＜0.001** |
| Arm pain | 1[Reference] | **1.259(1.045-1.517)** | **1.728(1.429-2.089)** | **1.967(1.592-2.429)** | **2.079(1.599-2.704)** | **3.436(2.569-4.595)** | **p＜0.001** |
| Wrist pain | 1[Reference] | **1.322(1.067-1.637)** | **1.748(1.406-2.174)** | **1.943(1.525-2.476)** | **2.151(1.598-2.894)** | **3.328(2.399-4.617)** | **p＜0.001** |
| Finger pain | 1[Reference] | 1.210(0.981-1.492) | **1.502(1.210-1.864)** | **1.524(1.192-1.947)** | **1.669(1.235-2.256)** | **2.822(2.035-3.912)** | **p＜0.001** |
| Chest pain | 1[Reference] | **1.342(1.055-1.707)** | **1.479(1.150-1.901)** | **1.808(1.375-2.376)** | **2.261(1.639-3.121)** | **3.356(2.356-4.782)** | **p＜0.001** |
| Stomachache | 1[Reference] | **1.278(1.045-1.562)** | **1.337(1.081-1.652)** | **1.795(1.429-2.255)** | **2.10 (1.600-2.775)** | **2.807(2.052-3.841)** | **p＜0.001** |
| Back pain | 1[Reference] | **1.260(1.047-1.517)** | **1.396(1.149-1.696)** | **1.532(1.232-1.906)** | **2.111(1.630-2.732)** | **2.178(1.594-2.977)** | **p＜0.001** |
| Waist pain | 1[Reference] | **1.182(1.021-1.370)** | **1.504(1.291-1.752)** | **1.767(1.489-2.099)** | **2.175(1.759-2.690)** | **2.626(1.793-3.848)** | **p＜0.001** |
| Buttock pain | 1[Reference] | 1.063(0.825-1.368) | **1.540(1.195-1.983)** | **1.605(1.208-2.134)** | **1.494(1.037-2.152)** | **3.051(2.101-4.430)** | **p＜0.001** |
| Leg pain | 1[Reference] | 1.137(0.968-1.336) | **1.499(1.270-1.770)** | **1.469(1.214-1.777)** | **1.762(1.393-2.228)** | **2.572(1.960-3.375)** | **p＜0.001** |
| Knee pain | 1[Reference] | 1.085(0.923-1.274) | **1.477(1.251-1.742)** | **1. 649(1.368-1.987)** | **1.816(1.439-2.291)** | **2.516(1.917-3.301)** | **p＜0.001** |
| Ankle pain | 1[Reference] | 1.170(0.935-1.464) | **1.642(1.310-2.058)** | **1.630(1.262-2.104)** | **2.262(1.682-3.042)** | **3.050(2.182-4.262)** | **p＜0.001** |
| Toe pain | 1[Reference] | 1.237(0.953-1.607) | **1.532(1.172-2.002)** | **1.354(0.992-1.847)** | **1.719(1.192-2.480)** | **2.310(1.532-3.484)** | **p＜0.001** |
| **Model 3^d^** |  |  |  |  |  |  |  |
| Headache | 1[Reference] | 1.175(0.993-1.390) | **1.397(1.173-1.665)** | **1.767(1.457-2.143)** | **1.877(1.476-2.386)** | **2.478(1.874-3.277)** | **p＜0.001** |
| Neck pain | 1[Reference] | 1.200(0.985-1.462) | **1.503(1.226-1.842)** | **1.748(1.394-2.192)** | **2.189(1.668-2.873)** | **3.049(2.239-4.152)** | **p＜0.001** |
| Shoulder pain | 1[Reference] | 1.115(0.943-1.318) | **1.391(1.169-1.654)** | **1.659(1.367-2.012)** | **1.926(1.518-2.443)** | **2.517(1.906-3.324)** | **p＜0.001** |
| Arm pain | 1[Reference] | **1.221(1.012-1.474)** | **1.610(1.328-2.951)** | **1.735(1.399-2.151)** | **1.814(1.388-2.371)** | **2.848(2.115-3.834)** | **p＜0.001** |
| Wrist pain | 1[Reference] | **1.274(1.027-1.579)** | **1.615(1.295-2.013)** | **1.693(1.323-2.167)** | **1.844(1.363-2.194)** | **2.687(1.922-3.756)** | **p＜0.001** |
| Finger pain | 1[Reference] | 1.210(0.981-1.492) | **1.617(1.305-2.002)** | **1.716(1.348-2.185)** | **1.925(1.431-2.590)** | **3.320(2.409-4.576)** | **p＜0.001** |
| Chest pain | 1[Reference] | **1.392(1.096-1.770)** | **1.575(1.228-2.021)** | **2.008(1.533-2.630)** | **2.562(1.865-3.519)** | **3.843(2.714-5.442)** | **p＜0.001** |
| Stomachache | 1[Reference] | **1.344(1.101-1.641)** | **1.461(1.185-1.802)** | **2.075(1.658-2.597)** | **2.487(1.899-3.259)** | **3.396(2.499-4.616)** | **p＜0.001** |
| Back pain | 1[Reference] | **1.315(1.093-1.581)** | **1.503(1.240-1.823)** | **1.733(1.398-2.149)** | **2.425(1.881-3.125)** | **2.568(1.889-3.490)** | **p＜0.001** |
| Waist pain | 1[Reference] | **1.238(1.070-1.432)** | **1.626(1.399-1.890)** | **2.012(1.701-2.381)** | **2.521(2.048-3.105)** | **3.345(2.606-4.293)** | **p＜0.001** |
| Buttock pain | 1[Reference] | 1.102(0.857-1.418) | **1.626(1.265-2.089)** | **1.762(1.331-2.333)** | **1.674(1.168-2.398)** | **2.946(2.025-4.286)** | **p＜0.001** |
| Leg pain | 1[Reference] | **1.184(1.009-1.389)** | **1.602(1.359-1.887)** | **1.647(1.366-1.987)** | **2.008(1.595-2.530)** | **2.981(2.283-3.892)** | **p＜0.001** |
| Knee pain | 1[Reference] | 1.130(0.963-1.326) | **1.581(1.342-1.861)** | **1.850(1.541-2.222)** | **2.073(1.650-2.604)** | **2.927(2.243-3.822)** | **p＜0.001** |
| Ankle pain | 1[Reference] | 1.221(0.977-1.526) | **1.760(1.408-2.202)** | **1.834(1.425-2.359)** | **2.588(1.934-3.464)** | **3.545(2.553-4.924)** | **p＜0.001** |
| Toe pain | 1[Reference] | **1.300(1.002-1.686)** | **1.668(1.279-2.175)** | **1.558(1.146-2.117)** | **2.038(1.421-2.923)** | **2.787(1.861-4.174)** | **p＜0.001** |
| **Model 4^e^** |  |  |  |  |  |  |  |
| Headache | 1[Reference] | 1.132(0.953-1.344) | **1.233(1.030-1.477)** | **1.528(1.253-1.864)** | **1.608(1.255-2.061)** | **1.954(1.462-2.613)** | **p＜0.001** |
| Neck pain | 1[Reference] | 1.107(0.903-1.356) | 1.231(0.996-1.520) | **1.338(1.056-1.695)** | **1.647(1.238-2.191)** | **2.083(1.502-2.889)** | **p＜0.001** |
| Shoulder pain | 1[Reference] | 1.063(0.896-1.262) | **1.219(1.019-1.457)** | **1.413(1.158-1.725)** | **1.637(1.280-2.094)** | **1.955(1.463-2.611)** | **p＜0.001** |
| Arm pain | 1[Reference] | 1.170(0.965-1.417) | **1.426(1.171-1.737)** | **1.486(1.192-1.853)** | **1.527(1.160-2.011)** | **2.215(1.628-3.015)** | **p＜0.001** |
| Wrist pain | 1[Reference] | 1.213(0.974-1.512) | **1.399(1.116-1.753)** | **1.407(1.092-1.812)** | **1.506(1.103-2.058)** | **1.982(1.400-2.807)** | **p＜0.001** |
| Finger pain | 1[Reference] | 1.150(0.928-1.425) | **1.300(1.042-1.624)** | 1.262(0.981-1.624) | **1.373(1.006-1.873)** | **2.171(1.546-3.050)** | **p＜0.001** |
| Chest pain | 1[Reference] | **1.307(1.022-1.671)** | 1.288(0.995-1.668) | **1.521(1.148-2.014)** | **1.940(1.392-2.705)** | **2.613(1.806-3.780)** | **p＜0.001** |
| Stomachache | 1[Reference] | 1.223(0.992-1.508) | 1.114(0.892-1.391) | **1.479(1.164-1.879)** | **1.694(1.266-2.267)** | **2.022(1.448-2.824)** | **p＜0.001** |
| Back pain | 1[Reference] | **1.212(1.002-1.467)** | 1.221(0.999-1.493) | **1.293(1.032-1.620)** | **1.796(1.375-2.348)** | **1.657(1.197-2.294)** | **p＜0.001** |
| Waist pain | 1[Reference] | 1.138(0.979-1.324) | **1.345(1.149-1.574)** | **1.538(1.288-1.836)** | **1.880(1.509-2.342)** | **2.276(1.746-2.968)** | **p＜0.001** |
| Buttock pain | 1[Reference] | 1.004(0.776-1.298) | **1.323(1.021-1.714)** | 1.325(0.991-1.773) | 1.206(0.830-1.752) | **1.922(1.295-2.854)** | **p=0.001** |
| Leg pain | 1[Reference] | 1.083(0.918-1.278) | **1.318(1.110-1.565)** | **1.237(1.016-1.507)** | **1.486(1.165-1.896)** | **2.000(1.505-2.657)** | **p＜0.001** |
| Knee pain | 1[Reference] | 1.026(0.868-1.213) | **1.280(1.077-1.521)** | **1.379(1.135-1.675)** | **1.501(1.176-1.915)** | **1.899(1.425-2.531)** | **p＜0.001** |
| Ankle pain | 1[Reference] | 1.101(0.875-1.385) | **1.405(1.113-1.774)** | **1.333(1.024-1.735)** | **1.846(1.357-2.510)** | **2.250(1.585-3.193)** | **p＜0.001** |
| Toe pain | 1[Reference] | 1.194(0.915-1.558) | **1.332(1.013-1.752)** | 1.126(0.820-1.548) | 1.429(0.982-2.080) | **1.751(1.145-2.678)** | **p=0.018** |

**Abbreviations:** ACE, Adverse Childhood Experience; OR, Odds Ratio.

**^a^** Model 1 was the crude model

**^b^** Reference: No ACE exposure

**^c^** Model 2 was adjusted for age, sex, body mass index

**^d^** Model 3 was adjusted for age, sex, body mass index, area of residence, educational level, childhood family economic hardship, smoking and drinking status, marital status, physical activity

**^e^** Model 4 was adjusted for age, sex, body mass index, area of residence, educational level, childhood family economic hardship, smoking and drinking status, marital status, physical activity and 14 chronic diseases (Hypertension, dyslipidemia, diabetes, heart disease, stroke, chronic lung disease, asthma, liver disease, cancer, digestive disease, kidney disease, arthritis, psychiatric disease, and memory-related disease)

**Table S3 Associations Between the Number of ACEs and Single Pain in the Overall Study Population and Subgroups**

|  | **OR (95% CI) by No. of ACEs** | | | | | | **P value for trend** | **P value for interaction** |
| --- | --- | --- | --- | --- | --- | --- | --- | --- |
| **Characteristic^a^** | **0=2288** | **1=3104** | **2=2266** | **3=1315** | **4=605** | **≥5=345** |  |  |
| **Overall study population** | 1 [Reference]^b^ | 1.101(0.838-1.446) | 1.213(0.906-1.625) | 1.176(0.830-1.668) | **1.947(1.297-2.925)** | **2.396(1.453-3.952)** | **p<0.001** |  |
| Subgroup |  |  |  |  |  |  |  |  |
| **Sex** |  |  |  |  |  |  |  |  |
| Male | 1 [Reference] | **1.878(1.216-2.900)** | **1.757(1.108-2.787)** | **1.981(1.185-3.312)** | **2.787(1.524-5.097)** | **3.899(1.927-7.889)** | **p<0.001** | 0.086 |
| Female | 1 [Reference] | 0.736(0.508-1.066) | 0.980(0.660-1.456) | 0.803(0.480-1.344) | 1.644(0.927-2.916) | 1.969(0.928-4.178) | p=0.089 |  |
| **Age** |  |  |  |  |  |  |  |  |
| <60 | 1 [Reference] | 1.060(0.690-1.631) | 1.204(0.760-1.907) | 1.180(0.680-2.046) | **2.394(1.306-4.387)** | **2.459(1.147-5.270)** | **p=0.004** | 0.250 |
| ≥60 | 1 [Reference] | 1.116(0.783-1.591) | 1.215(0.830-1.778) | 1.192(0.757-1.878) | 1.703(0.974-2.976) | **2.536(1.295-4.964)** | **p=0.010** |  |
| **Childhood family economic hardship** |  |  |  |  |  |  |  |  |
| No | 1 [Reference] | 1.052(0.754-1.467) | 1.176(0.813-1.701) | 1.345(0.855-2.116) | **2.062(1.169-3.637)** | **2.403(1.033-5.591)** | **p=0.004** | 0.720 |
| Yes | 1 [Reference] | 1.199(0.738-1.949) | 1.292(0.788-2.118) | 1.031(0.588-1.808) | **1.922(1.042-3.547)** | **2.559(1.305-5.016)** | **p=0.013** |  |
| **Area of residence** |  |  |  |  |  |  |  |  |
| Rural | 1 [Reference] | 1.045(0.775-1.408) | 1.183(0.860-1.626) | 1.020(0.690-1.509) | 1.587(0.996-2.529) | **1.953(1.101-3.463)** | **p=0.024** | **0.020** |
| Urban | 1 [Reference] | 1.377(0.695-2.730) | 1.482(0.700-3.138) | 2.198(0.986-4.902) | **4.962(2.033-12.109)** | **6.488(2.158-19.506)** | **p<0.001** |  |
| **Educational level completed** |  |  |  |  |  |  |  |  |
| None | 1 [Reference] | 0.974(0.554-1.713) | 1.263(0.691-2.310) | 0.658(0.280-1.545) | 2.205(0.932-5.217) | 1.677(0.514-5.471) | p=0.265 | 0.222 |
| Home School to Primary School | 1 [Reference] | 1.372(0.883-2.133) | 1.132(0.694-1.846) | 1.519(0.898-2.571) | 1.658(0.852-3.224) | **3.019(1.453-6.276)** | **p=0.014** |  |
| Middle school or above | 1 [Reference] | 0.902(0.569-1.427) | 1.297(0.813-2.068) | 1.189(0.657-2.152) | **2.369(1.220-4.602)** | **2.454(1.012-5.952)** | **p=0.004** |  |
| **Physical activities** |  |  |  |  |  |  |  |  |
| No activity | 1 [Reference] | 1.166(0.434-3.133) | **2.596(1.006-6.699)** | 1.260(0.370-4.285) | 2.405(0.542-10.682) | 1.130(0.109-11.741) | p=0.223 | 0.641 |
| With activity | 1 [Reference] | 1.081(0.813-1.438) | 1.125(0.825-1.535) | 1.177(0.817-1.696) | **1.886(1.234-2.883)** | **2.446(1.461-4.096)** | **p<0.001** |  |

**Abbreviations:** ACE, Adverse Childhood Experience; OR, Odds Ratio.

**^a^** The model was adjusted was adjusted for age, sex, body mass index, area of residence, educational level, childhood family economic hardship, smoking and drinking status, marital status, physical activity, except for the stratified variables in each subgroup.

**^b^** Reference: No ACE exposure.

**Table S4**  **Association****s Between the Number of ACEs and Multiple Pain in the Overall Study Population and Subgroups**

|  | **OR (95% CI) by No. of ACEs** | | | | | | ***P*-value for trend** | ***P*-value for interaction** |
| --- | --- | --- | --- | --- | --- | --- | --- | --- |
| **Characteristic^a^** | **0=2288** | **1=3104** | **2=2266** | **3=1315** | **4=605** | **≥5=345** |  |  |
| **Overall study population** | 1 [Reference]^b^ | **1.167(1.013-1.345)** | **1.368(1.178-1.588)** | **1.623(1.371-1.921)** | **2.064(1.666-2.557)** | **2.839(2.178-3.700)** | **p<0.001** |  |
| Subgroup |  |  |  |  |  |  |  |  |
| **Sex** |  |  |  |  |  |  |  |  |
| Male | 1 [Reference] | 1.205(0.964-1.505) | 1.190(0.940-1.505) | **1.624(1.257-2.099)** | **2.009(1.457-2.770)** | **3.363(2.302-4.912)** | **p<0.001** | 0.802 |
| Female | 1 [Reference] | 1.144(0.952-1.375) | **1.535(1.263-1.865)** | **1.605(1.280-2.012)** | **2.098(1.570-2.803)** | **2.437(1.683-3.530)** | **p<0.001** |  |
| **Age** |  |  |  |  |  |  |  |  |
| <60 | 1 [Reference] | 1.186(0.950-1.480) | **1.514(1.202-1.909)** | **1.785(1.374-2.318)** | **2.091(1.491-2.934)** | **3.137(2.121-4.639)** | **p<0.001** | 0.216 |
| ≥60 | 1 [Reference] | 1.147(0.954-1.380) | **1.267(1.041-1.542)** | **1.530(1.225-1.912)** | **2.087(1.580-2.758)** | **2.622(1.821-3.774）** | **p<0.001** |  |
| **Childhood family economic hardship** |  |  |  |  |  |  |  |  |
| No | 1 [Reference] | 1.148(0.961-1.372) | **1.376(1.133-1.670)** | **1.809(1.437-2.277)** | **2.291(1.675-3.132)** | **4.222(2.760-6.458)** | **p<0.001** | **0.038** |
| Yes | 1 [Reference] | 1.178(0.929-1.493) | **1.345(1.059-1.709)** | **1.440(1.114-1.862)** | **1.866(1.375-2.533)** | **2.227(1.565-3.167)** | **p<0.001** |  |
| **Area of** **residence** |  |  |  |  |  |  |  |  |
| Rural | 1 [Reference] | 1.162(0.992-1.361) | **1.332(1.127-1.574)** | **1.633(1.353-1.970)** | **2.030(1.600-2.577)** | **2.672(1.983-3.601)** | **p<0.001** | 0.570 |
| Urban | 1 [Reference] | 1.173(0.851-1.617) | **1.549(1.108-2.167)** | **1.658(1.119-2.456)** | **2.391(1.459-3.918)** | **3.424(1.902-6.165)** | **p<0.001** |  |
| **Educational level completed** |  |  |  |  |  |  |  |  |
| None | 1 [Reference] | 1.026(0.766-1.375) | **1.414(1.042-1.920)** | 1.365(0.963-1.935) | **2.557(1.630-4.011)** | **2.191(1.233-3.894)** | **p<0.001** | 0.678 |
| Home School to Primary School | 1 [Reference] | 1.169(0.939-1.456) | **1.319(1.048-1.660)** | **1.623(1.260-2.091)** | **1.613(1.168-2.226)** | **2.808(1.912-4.125)** | **p<0.001** |  |
| Middle school or above | 1 [Reference] | 1.264(0.989-1.616) | **1.427(1.097-1.856)** | **1.903(1.404-2.579)** | **2.626(1.788-3.856)** | **3.416(2.111-5.530)** | **p<0.001** |  |
| **Physical activities** |  |  |  |  |  |  |  |  |
| No activity | 1 [Reference] | 1.375(0.895-2.114) | **1.887(1.200-2.968)** | **1.865(1.112-3.127)** | **1.865(1.672-7.115)** | **4.623(1.874-11.406)** | **p<0.001** | 0.193 |
| With activity | 1 [Reference] | 1.142(0.983-1.327) | **1.317(1.124-1.544)** | **1.598(1.336-1.911)** | **1.963(1.566-2.459)** | **2.668(2.018-3.528)** | **p<0.001** |  |

**Abbreviations:** ACE, Adverse Childhood Experience; OR, Odds Ratio.

**^a^** The model was adjusted was adjusted for age, sex, body mass index, area of residence, educational level, childhood family economic hardship, smoking and drinking status, marital status, physical activity, except for the stratified variables in each subgroup.

**^b^** Reference: No ACE exposure.

**Figure S1. Associations Between Individual ACE Indicator and Single pain.**

**
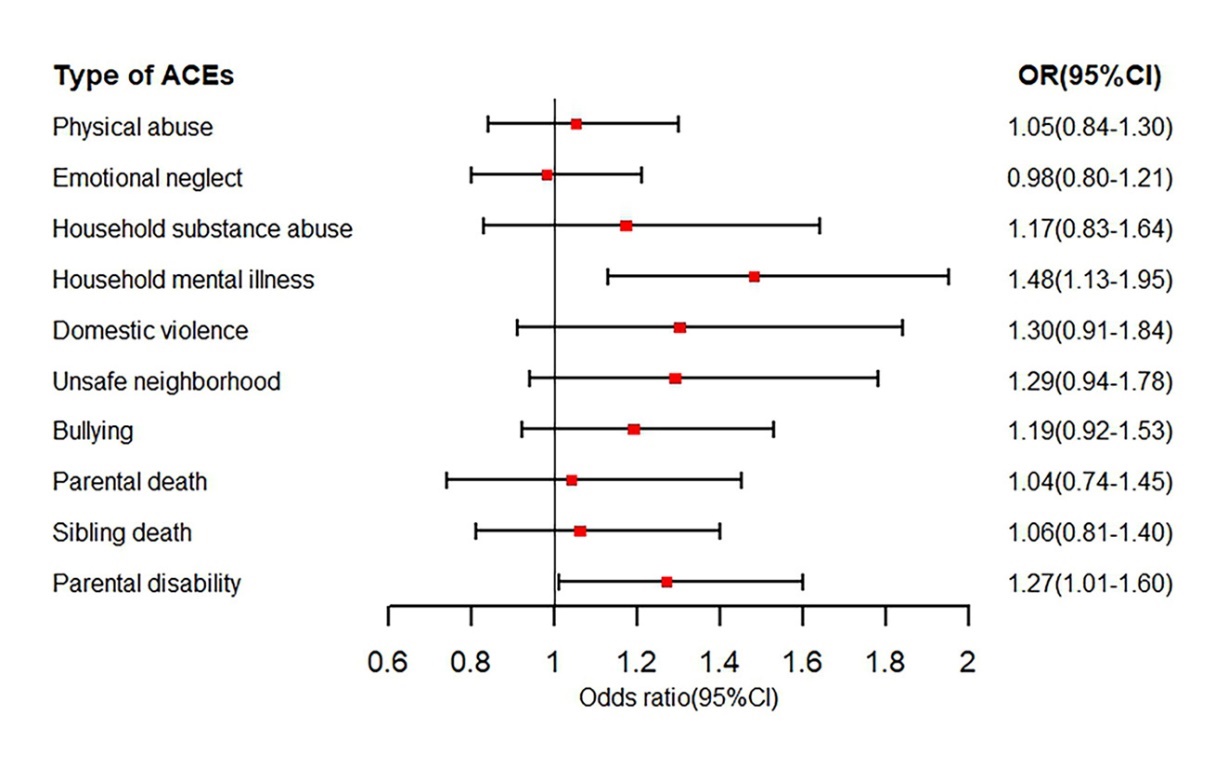
**

**Figure S2. Associations Between Individual ACE Indicator and multiple pain.**

**
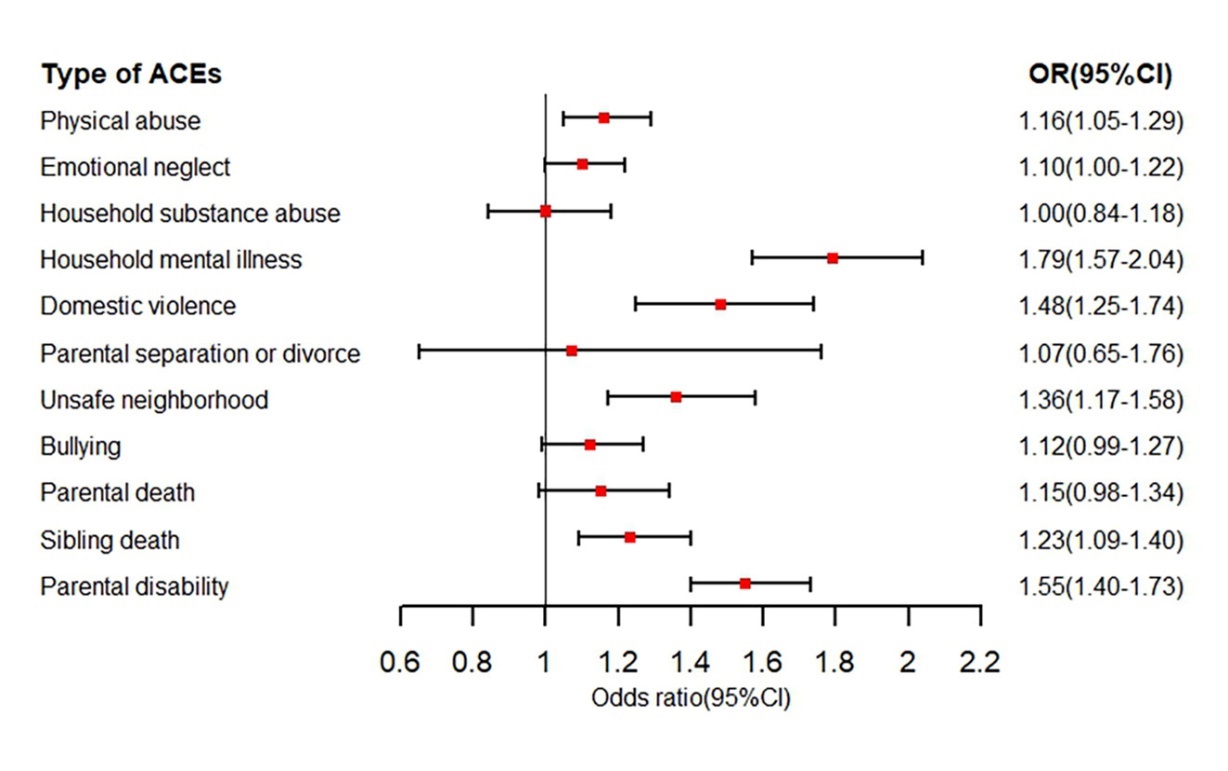
**

**Figure S3. Associations Between Individual ACE Indicator and Headache**

**
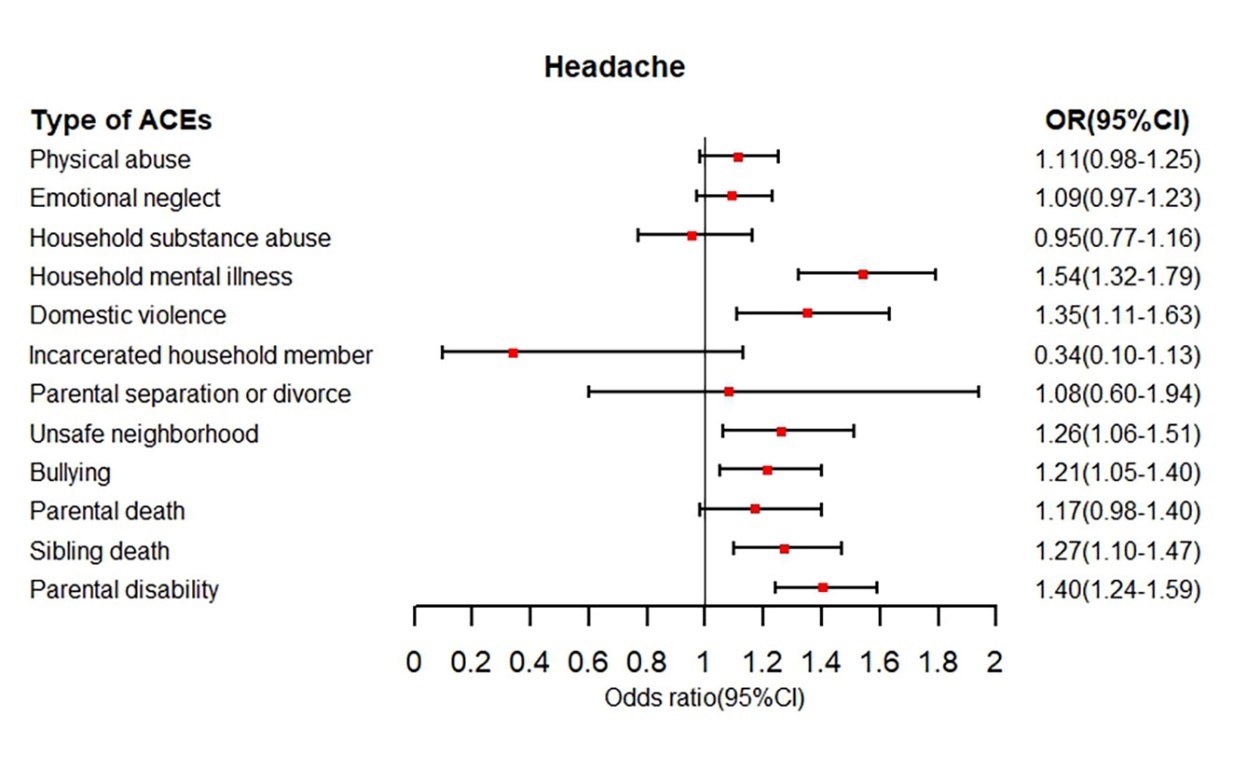
**

**Figure S4. Associations Between Individual ACE Indicator and Neck Pain**

**
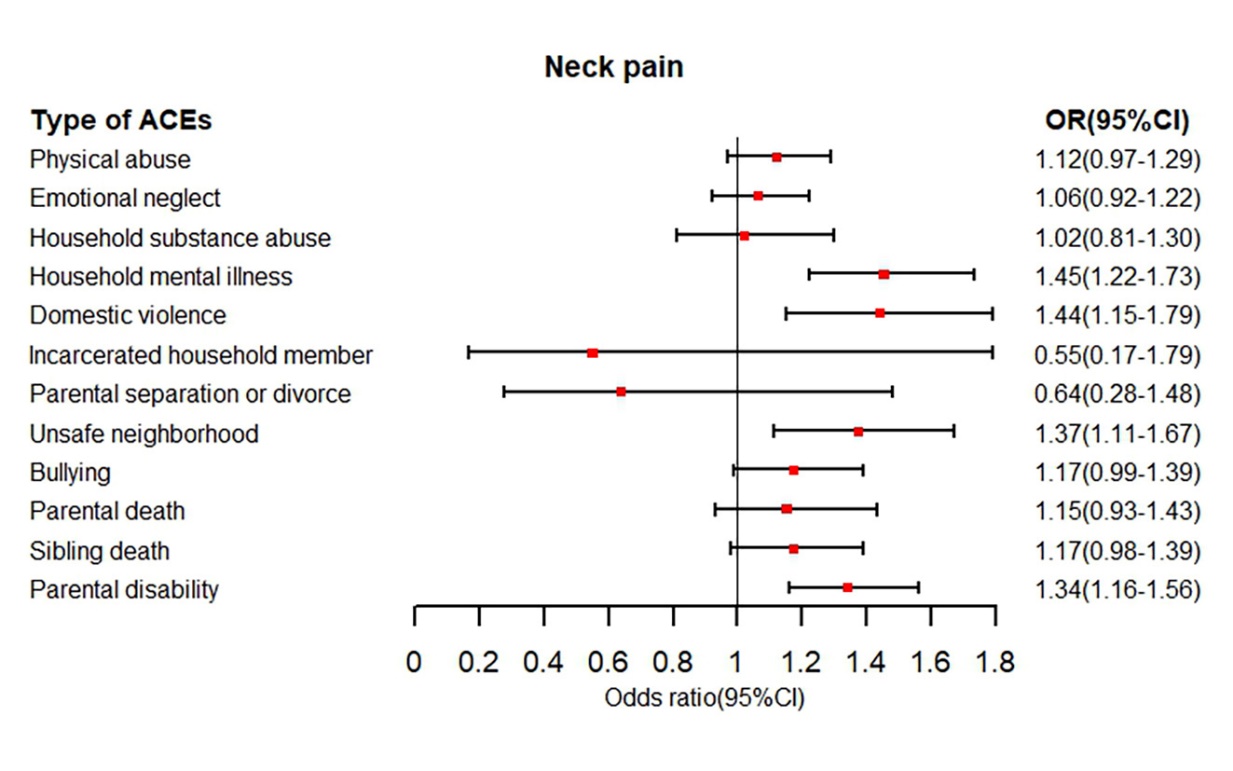
**

**Figure S5. Associations Between Individual ACE Indicator and Shoulder Pain**

**
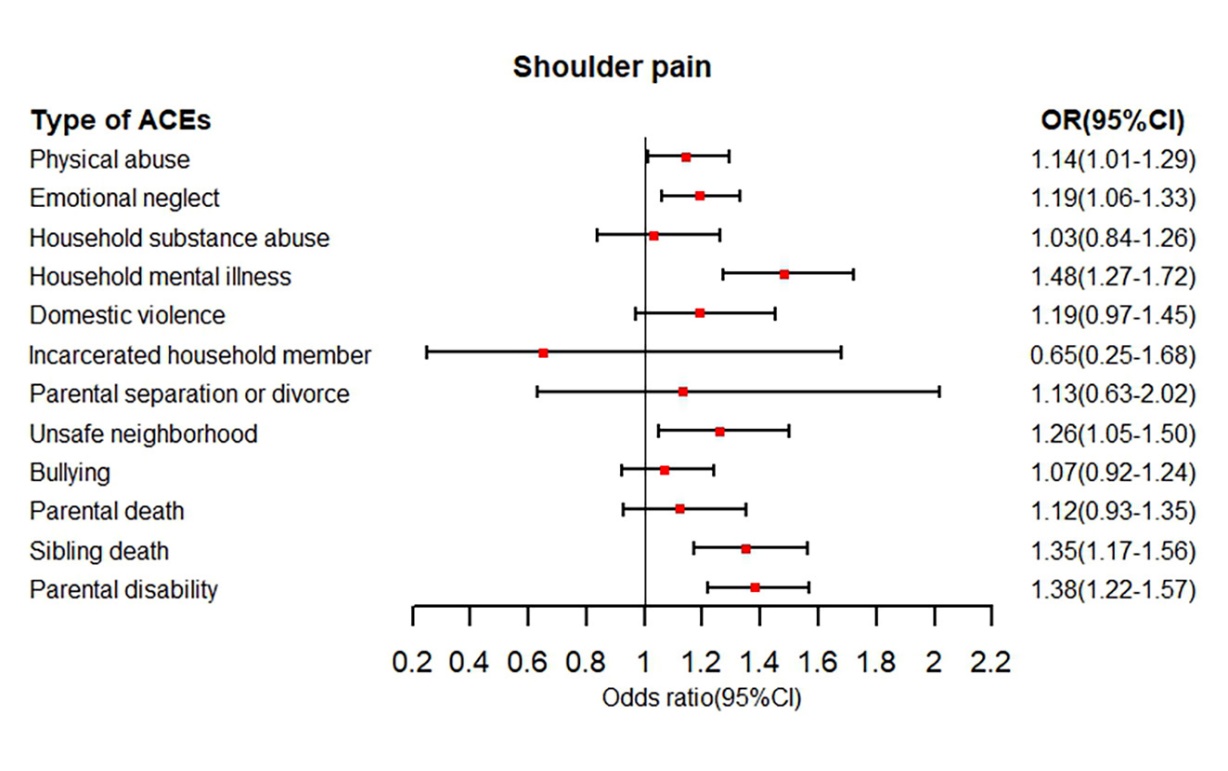
**

**Figure S6. Associations Between Individual ACE Indicator and Arm Pain**


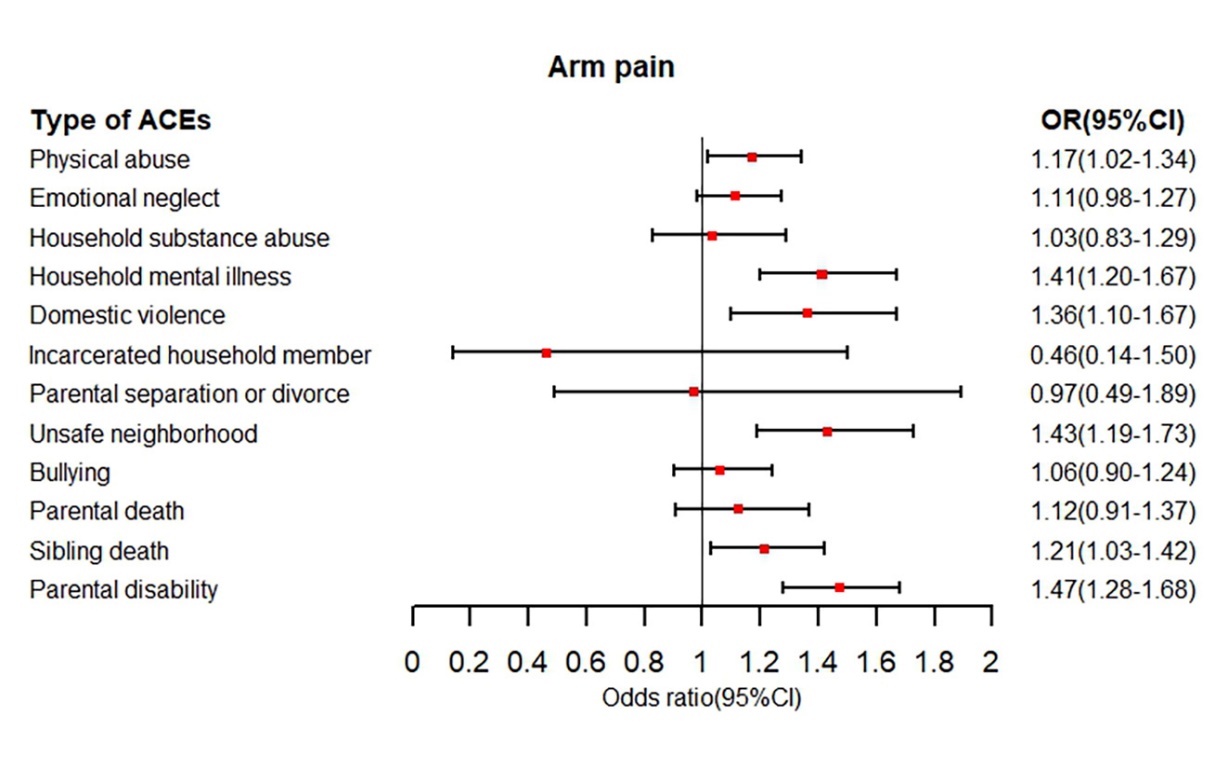


**Figure S7. Associations Between Individual ACE Indicator and Wrist Pain**

**
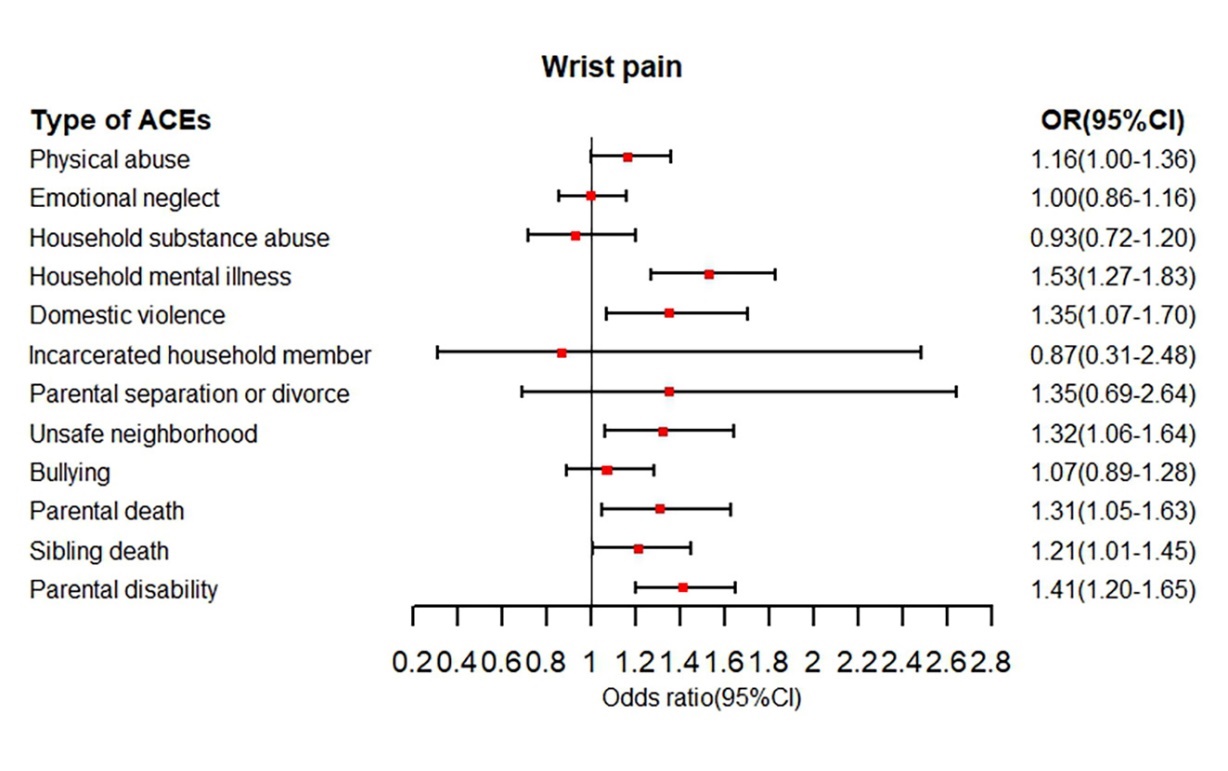
**

**Figure S8. Associations Between Individual ACE Indicator and Finger Pain**

**
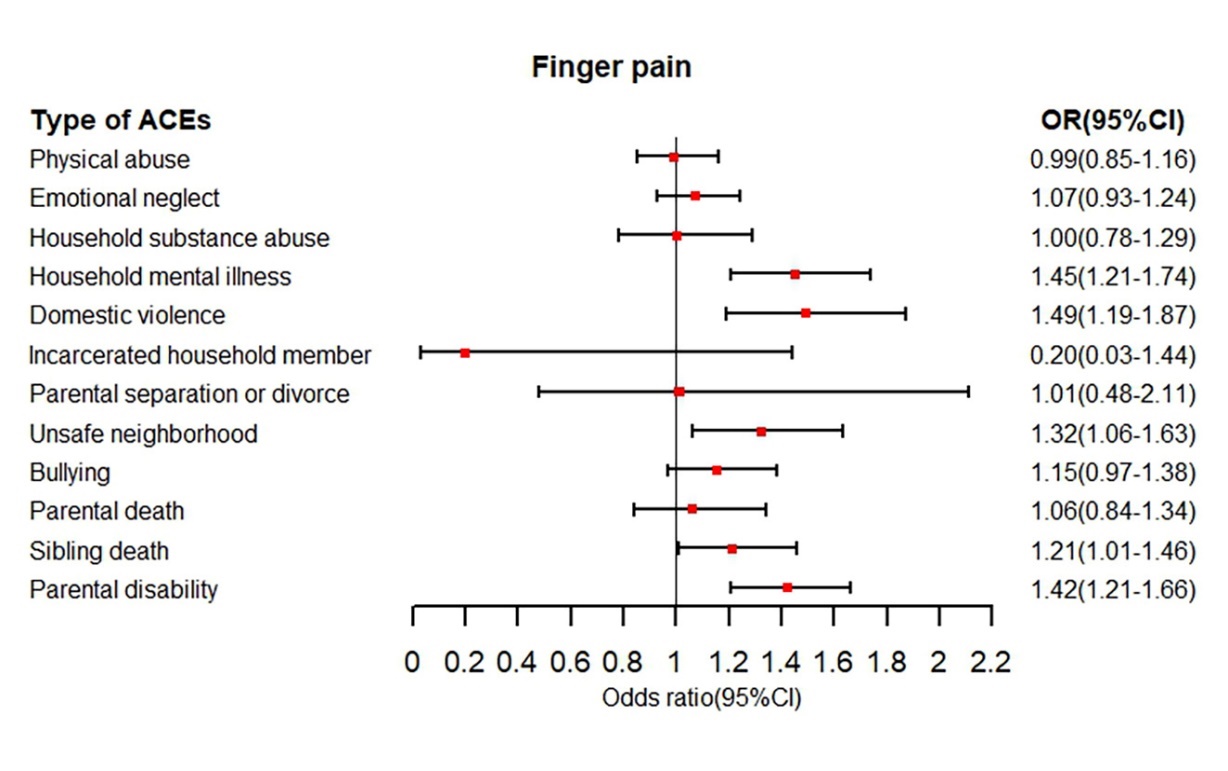
**

**Figure S9. Associations Between Individual ACE Indicator and Chest Pain**

**
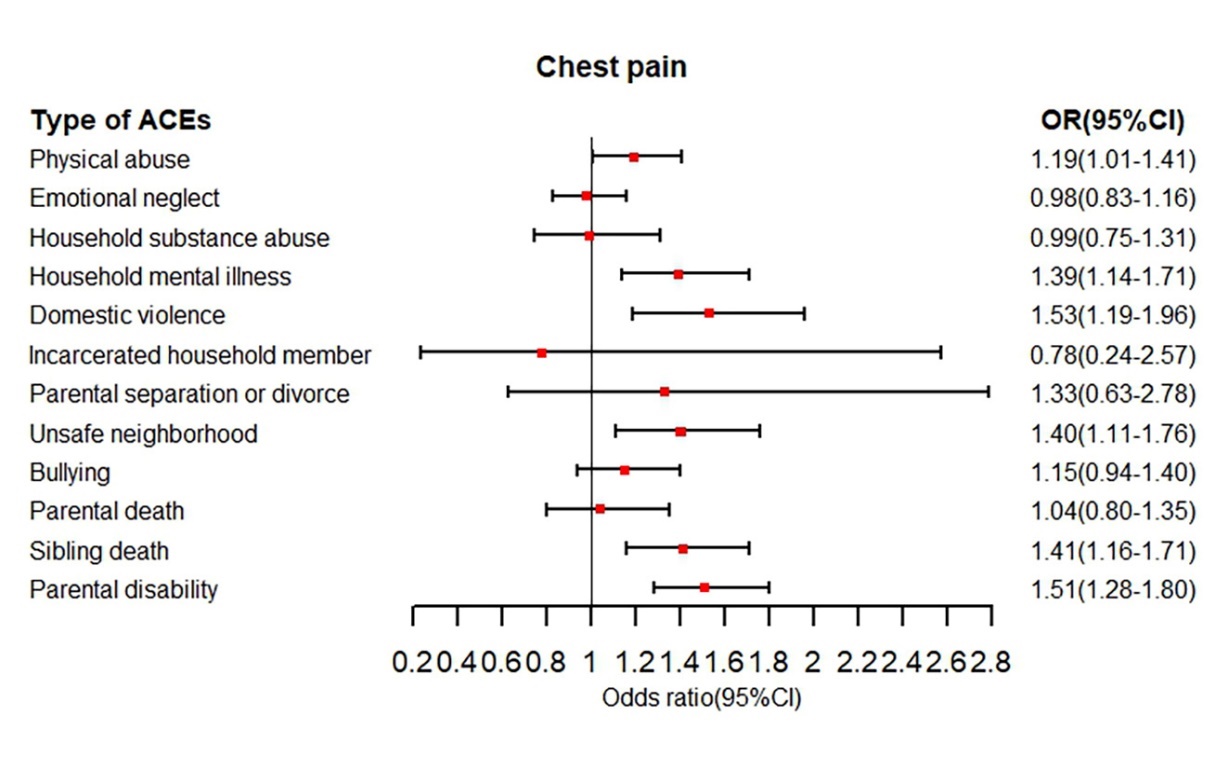
**

**Figure S10. Associations Between Individual ACE Indicator and Stomachache**

**
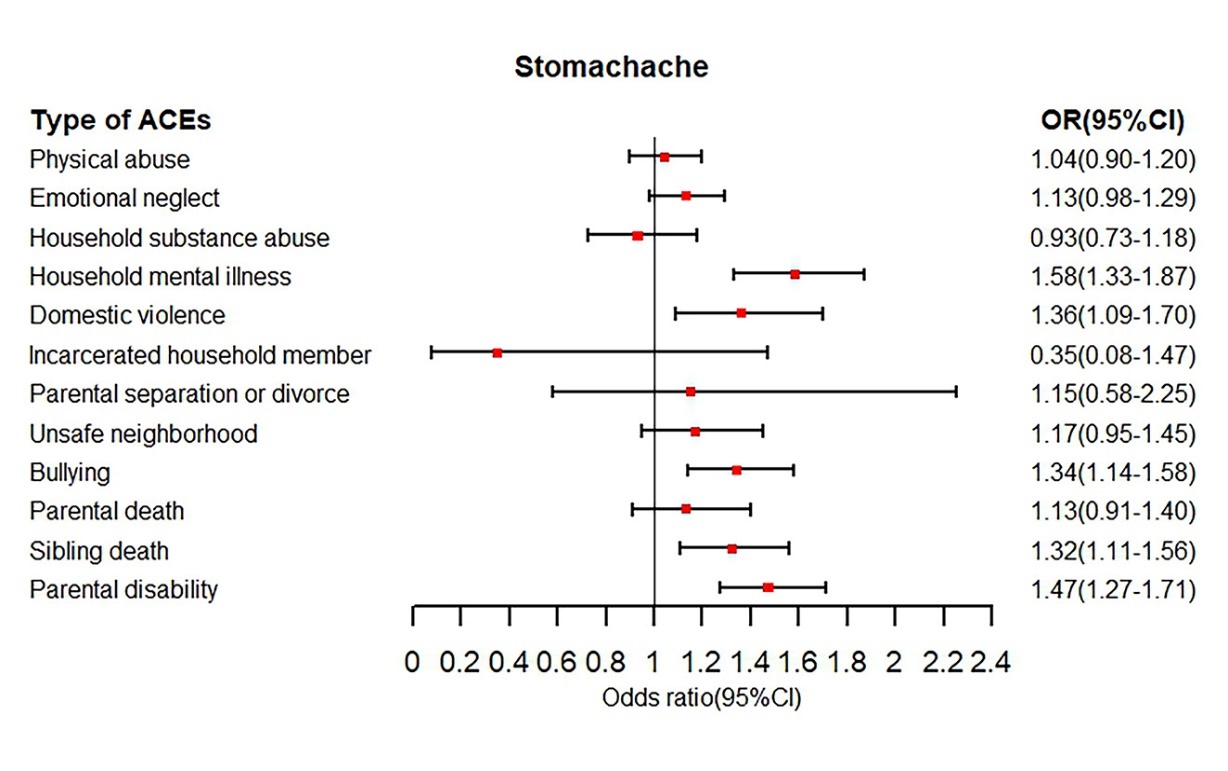
**

**Figure S11. Associations Between Individual ACE Indicator and Back Pain**

**
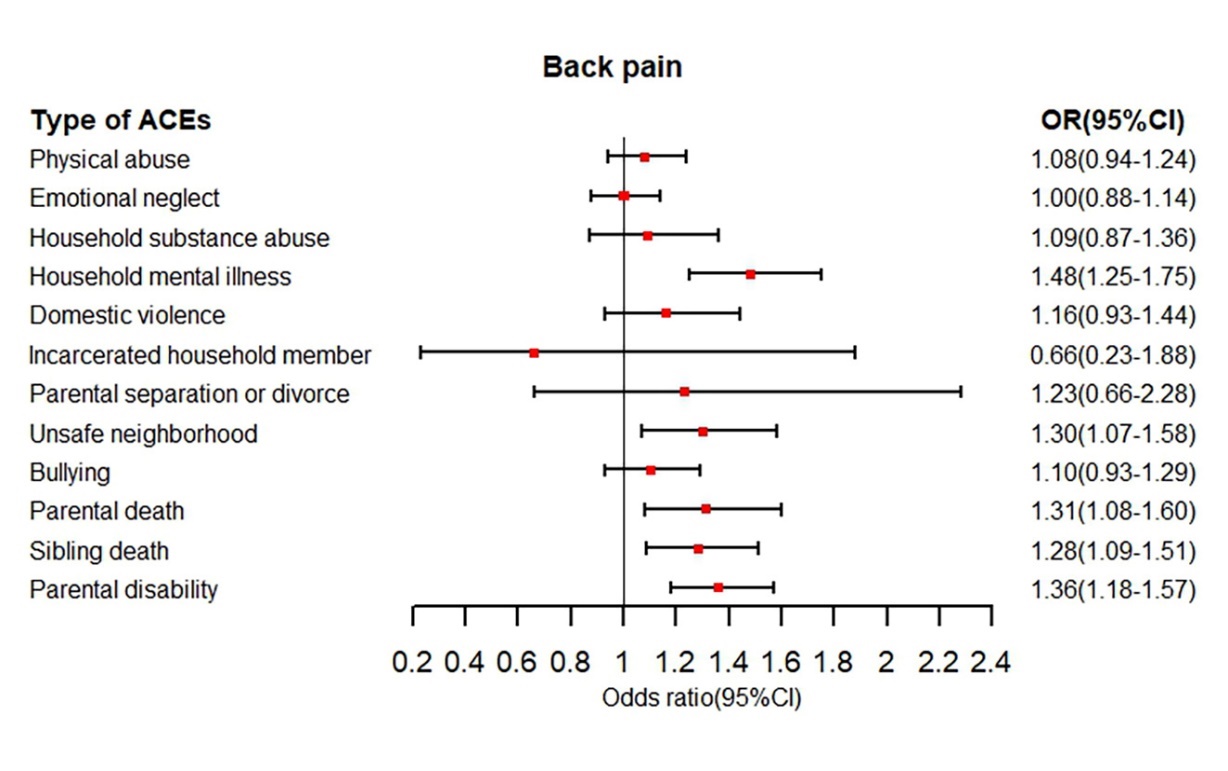
**

**Figure S12. Associations Between Individual ACE Indicator and Waist Pain**

**
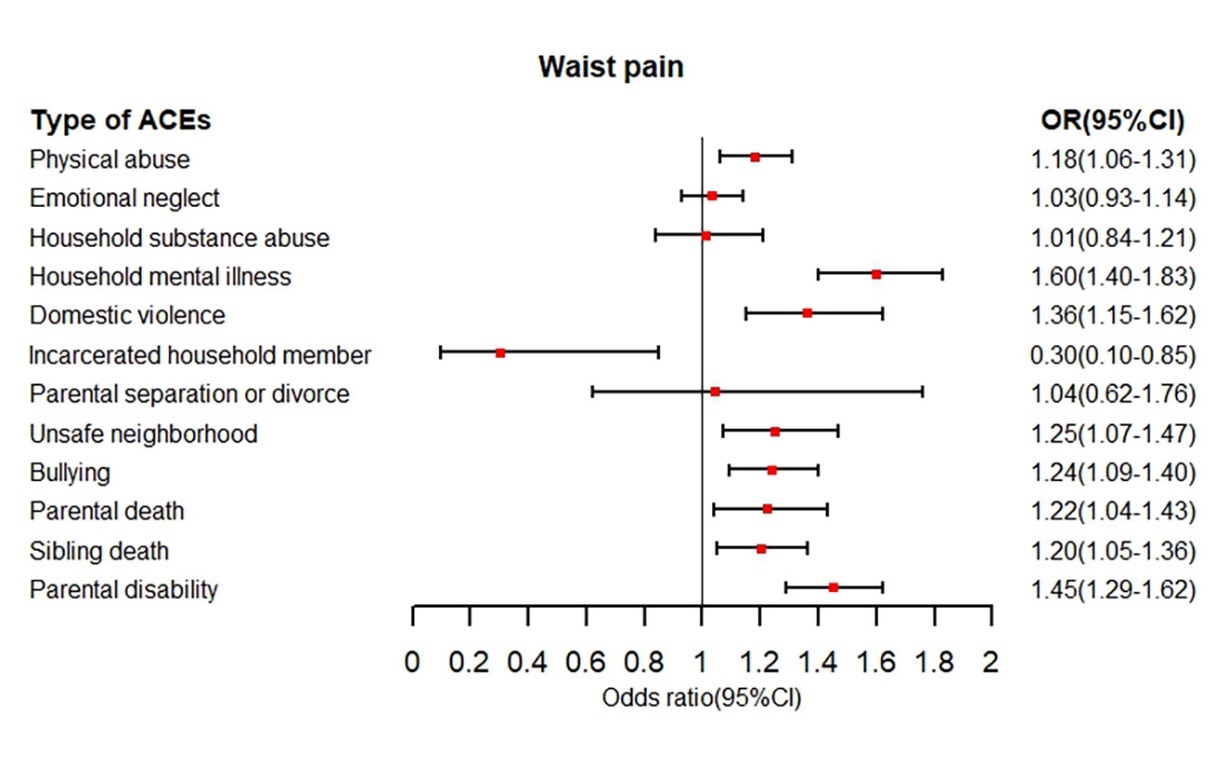
**

**Figure S13. Associations Between Individual ACE Indicator and Bottock Pain**

**
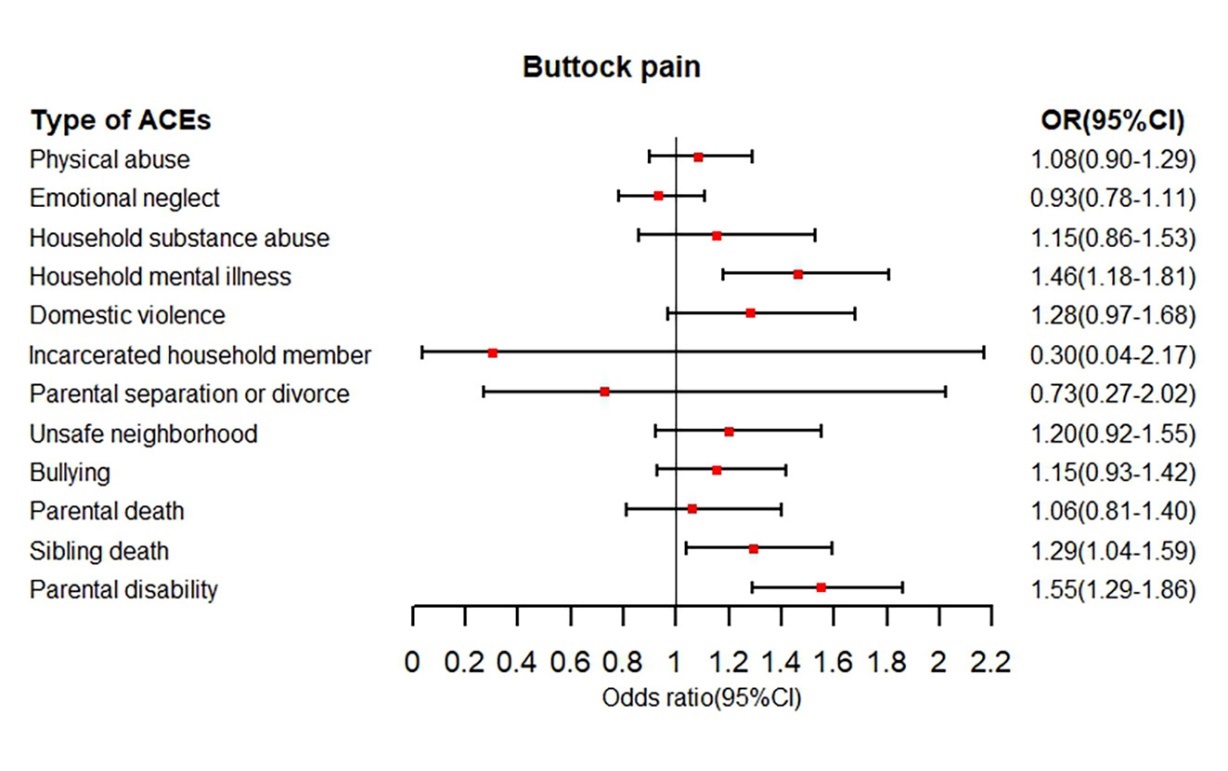
**

**Figure S14. Associations Between Individual ACE Indicator and Leg Pain**

**
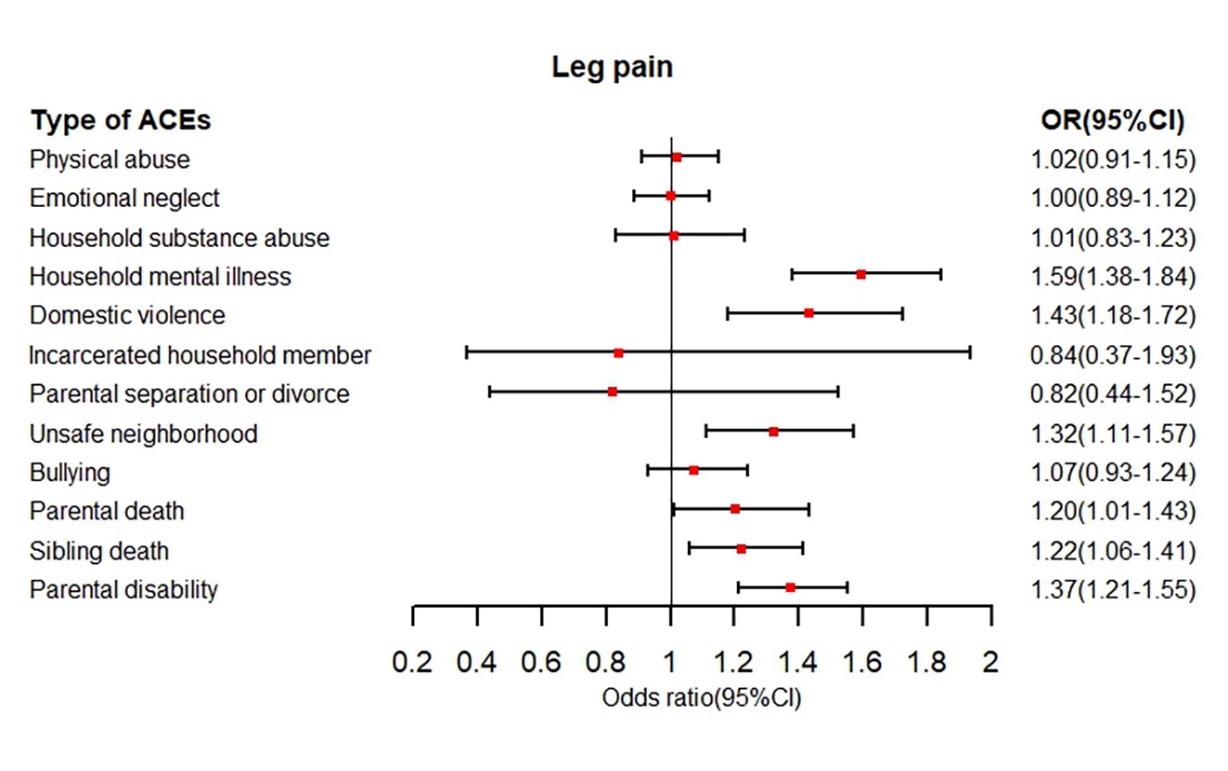
**

**Figure S15. Associations Between Individual ACE Indicator and Knee Pain**

**
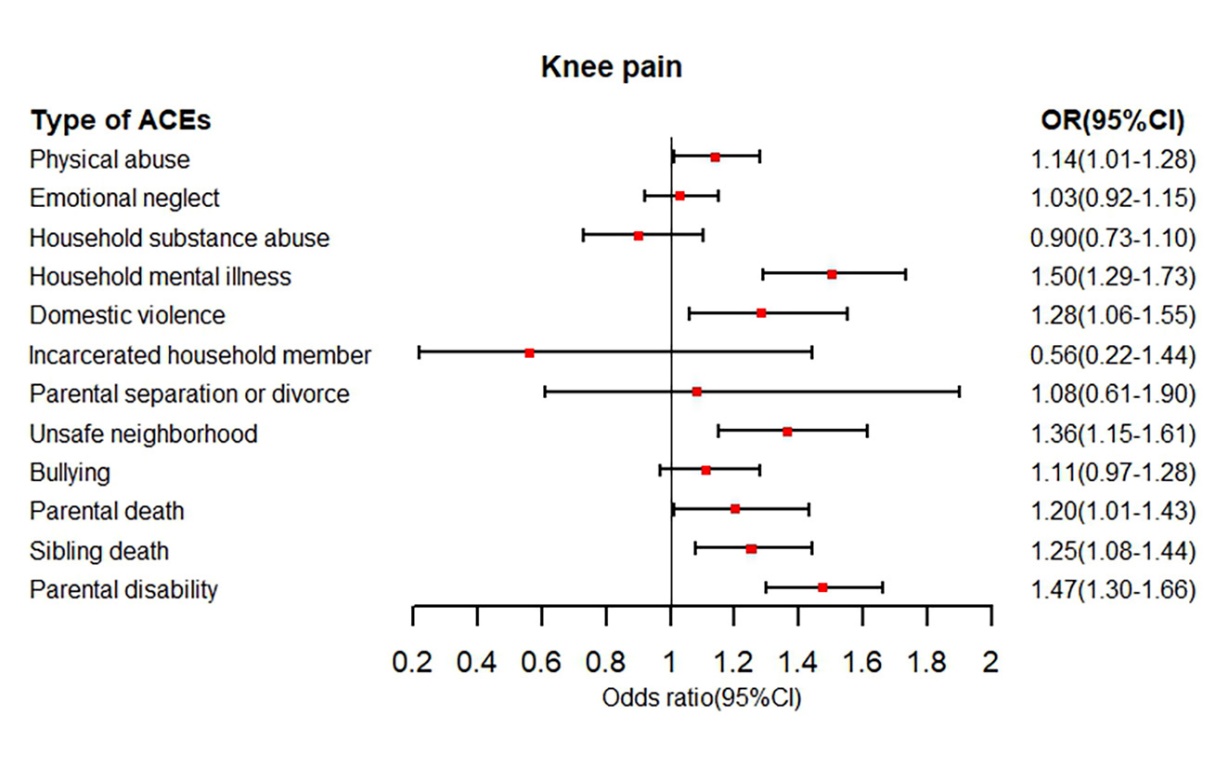
**

**Figure S16. Associations Between Individual ACE Indicator and Ankle Pain**

**
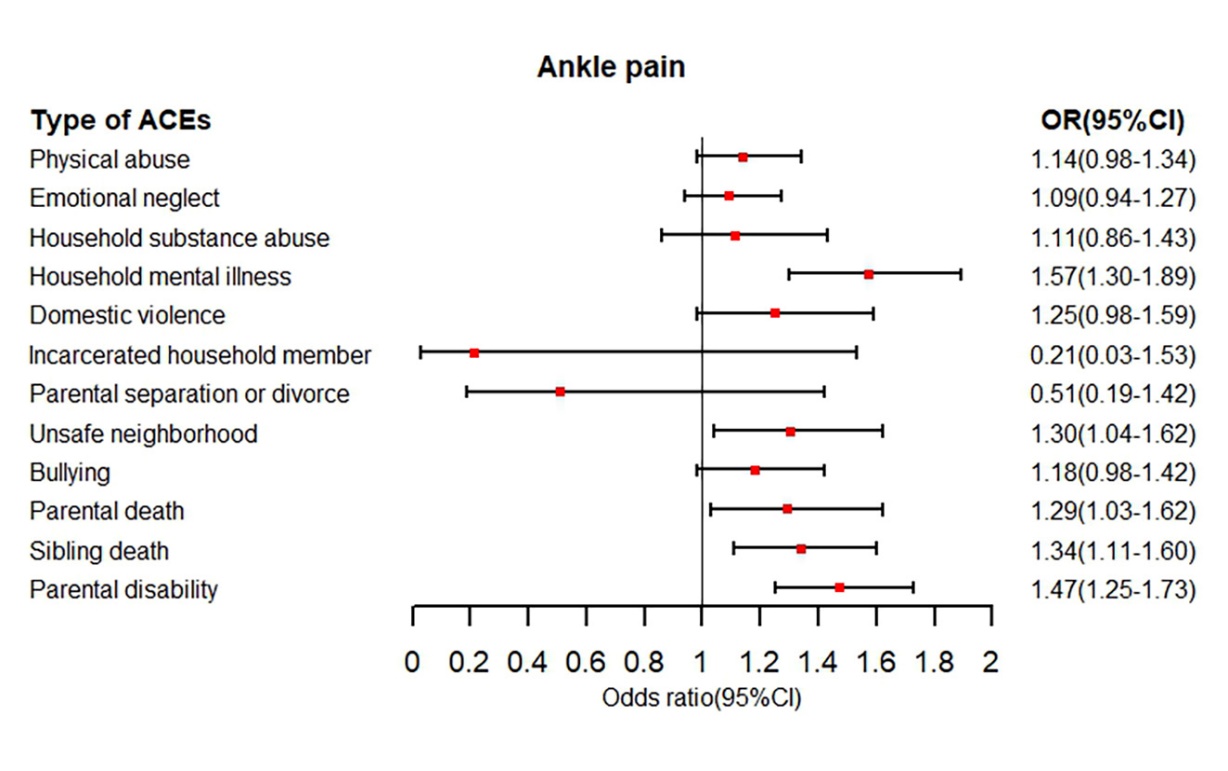
**

**Figure S17. Associations Between Individual ACE Indicator and Toe Pain**


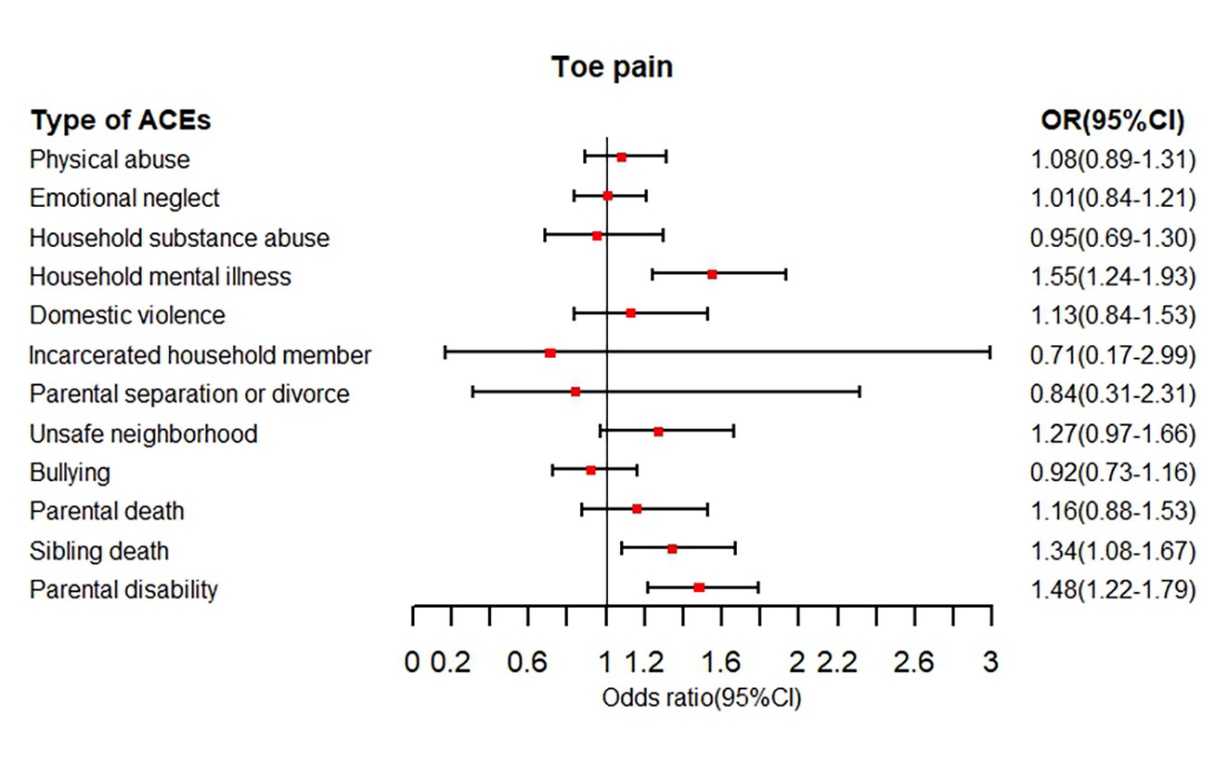


Model was adjusted for age, sex, body mass index, area of residence, educational level, childhood family economic hardship, smoking and drinking status, marital status, physical activity and 14 chronic diseases (hypertension, dyslipidemia, diabetes, heart disease, stroke, chronic lung disease, asthma, liver disease, cancer, digestive disease, kidney disease, arthritis, psychiatric disease, and memory-related disease).

**Reference**

1. Zhou BF. Predictive values of body mass index and waist circumference for risk factors of certain related diseases in Chinese adults--study on optimal cut-off points of body mass index and waist circumference in Chinese adults. Biomedical and environmental sciences : BES. 2002;15(1):83-96.

2. Lin L, Wang HH, Lu C, Chen W, Guo VY. Adverse Childhood Experiences and Subsequent Chronic Diseases Among Middle-aged or Older Adults in China and Associations With Demographic and Socioeconomic Characteristics. JAMA network open. 2021;4(10):e2130143.
